# CD47/SIRPα blocking peptide identification and synergistic effect with irradiation for cancer immunotherapy

**DOI:** 10.1136/jitc-2020-000905

**Published:** 2020-10-05

**Authors:** Hongfei Wang, Yixuan Sun, Xiuman Zhou, Chunxia Chen, Ling Jiao, Wanqiong Li, Shanshan Gou, Yanying Li, Jiangfeng Du, Guanyu Chen, Wenjie Zhai, Yahong Wu, Yuanming Qi, Yanfeng Gao

**Affiliations:** 1 School of Life Sciences, Zhengzhou University, Zhengzhou, Henan, China; 2 School of Pharmaceutical Sciences (Shenzhen), Sun Yat-sen University, Shenzhen, Guangdong, China

**Keywords:** antineoplastic protocols, phagocytosis, macrophages, immunotherapy, radiotherapy

## Abstract

**Background:**

Immunotherapy has achieved remarkable advances via a variety of strategies against tumor cells that evade immune surveillance. As important innate immune cells, macrophages play important roles in maintaining homeostasis, preventing pathogen invasion, resisting tumor cells and promoting adaptive immune response. CD47 is found to be overexpressed on tumor cells and act as a don’t eat me’ signal, which contributes to immune evasion. Macrophages mediated phagocytosis via blockade CD47/SIRPα (signal regulatory protein alpha) interaction was proved to induce effective antitumor immune response.

**Methods:**

A novel peptide pep-20, specifically targeting CD47 and blocking CD47/SIRPα interaction, was identified via high-throughput phage display library bio-panning. The capability to enhance the macrophage-mediated phagocytosis activities and antitumor effects of pep-20 were investigated. The mechanism of pep-20 to induce T-cell response was explored by ex vivo analysis and confirmed via macrophage depleting strategy. The structure-activity relationship and D-amino acid substitution of pep-20 were also studied. The antitumor effects and mechanism of a proteolysis resistant D-amino acid derivate pep-20-D12 combined with irradiation (IR) were also investigated.

**Results:**

Pep-20 showed remarkable enhancement of macrophage-mediated phagocytosis to both solid and hematologic tumor cells in vitro, and inhibited tumor growth in immune-competent tumor-bearing mice. Furthermore, pep-20 promoted macrophages to mobilize the antitumor T-cell response with minimal toxicity. Furthermore, systemic administration of the derivate pep-20-D12 showed robust synergistic antitumor efficacy in combination with IR.

**Conclusion:**

In summary, these results demonstrated that CD47/SIRPα blocking peptides, pep-20 and its derivate, could serve as promising candidates to promote macrophages-mediated phagocytosis and immune response in cancer immunotherapy.

## Background

As one of the most important components of innate immune, macrophages play key roles in recognizing and removing foreign, aged, damaged and dying cells from tissues and maintaining tissue homeostasis. Besides, macrophages can prevent the invasion of exogenous pathogens and eliminate endogenous pathogens to promote adaptive immune response.^1^ Macrophages-mediated phagocytosis is modulated via the balance of pro-phagocytosis and anti-phagocytosis signals, which expose on the surface of objective cells under normal physiological conditions. For example, macrophages can recognize specific ‘eat me’ signals, including phosphatidylserine, intercellular adhesion molecule 3 and calreticulin on the dying cells distinguished from the nearby normal cells via respective engulfment receptors. Subsequently, these dying cells are uptaken by macrophages. Conversely, tumor cells express the ‘don't eat me’ signals on the surface to suppress phagocytosis.^2 3^ Therefore, it is urgent to develop appropriate therapeutic agents to block the anti-phagocytosis signals and improve the antitumor therapeutic efficacy.

CD47 is well known to act as a ‘don’t eat me’ signal, interact with SIRPα on macrophages to deliver negative regulatory signals, and thus prevent phagocytic removal of the cells by the immune system.^4^ Previous studies have shown that upregulation of CD47 on hematopoietic stem cells and progenitor cells could act as a self-protective strategy against phagocytosis to avoid additional clearance during inflammation-mediated migration phase.^5^ CD47 might serve as a ‘marker of self’ on red blood cells, as CD47-deficient hematopoietic cells can be rapidly cleared from the bloodstream.^6 7^ Frustratingly, many studies have shown that overexpression of CD47 could be detected in many types of solid tumors and hematologic malignancies, including cancer stem cells.^8^ Tumor cells can upregulate CD47 as the ‘don't eat me’ signal, which in turn prevents themselves from being recognized and cleared by macrophages to escape immune surveillance. Fortunately, more studies have confirmed that CD47 blockade could reactivate the phagocytic efficacy of macrophages, and various strategies have been established for disrupting the CD47/SIRPα interaction. Among them, anti-CD47 antibodies can trigger macrophages-mediated phagocytosis and exhibit greater antitumor therapeutic efficacy against solid tumors or hematological malignancies. Moreover, SIRPα mutants ‘CV1’ and CD47 mutants ‘Velcro-CD47’ with high affinities have been reported to be adjuvants to augment the efficacy of therapeutic antibodies.^9 10^ For example, anti-human CD47 antibody (B6H12) can markedly suppress tumor growth in ovarian, breast, colon cancer, glioblastoma and acute lymphoblastic leukemia in NOD-scid Il2rγ null mice xenotransplantation models.^11^ Also, anti-CD47 antibody can synergize with rituximab (anti-CD20) to promote phagocytosis of macrophages and eliminate non-Hodgkin’s lymphoma.^12^ Furthermore, several studies suggested that blockade of CD47/SIRPα interaction could further activate the function of adaptive immune response towards cancer that is particularly mediated by cytotoxic T lymphocytes in immunocompetent mouse models.^13 14^ Anti-mouse CD47 antibody therapeutic efficacy can be impaired by depletion of CD8^+^ T cells, and the induced memory T-cell mediated response can completely resist tumor cells re-challenge.^15^ These findings confirmed that CD47 blockade have emerged as a promising strategy for cancer immunotherapy.

To date, agents targeting the CD47/SIRPα axis include antibodies and recombinant mutant fusion proteins which have been performed in preclinical studies and clinical trials, either as monotherapy or in combination with other therapeutics. These clinical trials were conducted for various indications in several solid tumors or hematological malignancies. However, the potent receptor occupation of these agents led to the potential side effects, such as anemia.^16^ Therefore, it is very urgent to develop CD47 blocking therapeutics which could reduce the potential side effects and maintain a good balance between safety and efficacy.

Peptides can retain the advantages of antibodies, which also have other merits such as lower immunogenicity, greater tissue penetration owing to their smaller size and minimize the risks of systemic toxicity. Moreover, peptides are easier to synthesize and can be modified by artificial methods with low cost. Peptide drugs have shown specific advantages and wide application prospects in inducing tumor cell apoptosis, inhibiting angiogenesis and being used as vaccines or drug carriers.^17 18^ The crystal structure of CD47/SIRPα indicated that receptor-ligand interactions occurred via the connection of amino acid residues of adjacent peptide segments. The screening strategy of phage-display library has been widely used to identify specific peptides which are capable of binding to corresponding targets, including enzymes, hormones, receptors or tissues.^19 20^ However, peptides targeting CD47 to block CD47/SIRPα interaction for cancer immunotherapy still remain to be investigated.

In this study, a subtractive phage bio-panning strategy was applied to identify peptides that possessed the specificity of binding to human CD47 and blocking the CD47/SIRPα interaction. The antitumor therapeutic effects and mechanism of the peptide candidates were investigated, as well as combination of the D-amino acid-modified peptide derivate with irradiation (IR).

## Methods

### Binding assays

Human or mouse CD47-IgV-Domain proteins were labeled with NT-647 dye following the microscale thermophoresis (MST) assay protocol, and the labeled protein used for each assay was about 50 to 200 nM. Unlabeled peptides or ligand protein solution was diluted for appropriate concentration serially in buffer (50 mM Tris, 150 mM NaCl, 10 mM MgCl_2_, 0.05% Tween-20, 0.5% DMSO and pH 7.5). The labeled proteins were then added to peptides or ligand protein solutions by 1∶1 (v/v). The samples were loaded into silica capillaries after incubation at room temperature for 30 min. Binding constants were determined by the MST assay according to manufacturer’s instructions with 20% or 40% light emitting diode (LED) power, 40% MST power at 25°C (Nano Temper, Monolith NT.115, Germany). Data analysis were performed by using a Nanotemper Analysis software.

### Blocking assays

Human or mouse CD47-IgV-Domain-hIg fusion proteins (20 nM, Sino Biological, China), or biotinylated human CD47 protein (20 nM, ACROBiosystems, USA) was incubated with titrating concentrations of peptides or anti-human CD47 antibody (B6H12) in phosphate-buffered saline (PBS, pH 7.4) for 1 hour at 4°C. The mixture was incubated with human or mice SIRPα overexpressed Chinese hamster ovary cells for 30 min at 4°C, washed to remove unbound mixtures and phycoerythrin (PE)-conjugated goat anti-human IgG or PE-conjugated streptavidin was incubated (eBioscience, USA). Cells were then analyzed by flow cytometry (BD Biosciences, USA). The IC_50_ was determined from sigmoidal dose response curves illustrated by GraphPad Prism.

### Phagocytosis assays in vitro

Murine bone marrow cells were isolated from 7 to 11 weeks old C57BL/6 or BALB/c mice. Human peripheral blood mononuclear cells were collected from venous blood of healthy volunteers, diluted with 2×PBS (pH 7.4) and separated with Ficoll density gradient. The cells were cultured in DMEM (GIBCO, USA) supplemented with 10% fetal bovine serum and 20 ng/mL granulocyte macrophage colony-stimulating factor (GM-CSF) or macrophage colony-stimulating factor (M-CSF, Peprotech, USA) for 7 days. Meanwhile, medium was replaced with fresh medium containing cytokine, and then the adherent cells were harvested. Phagocytosis assays were performed by co-culture of macrophages with carboxyfluorescein succinimidyl ester (CFSE^+^) or green fluorescent protein (GFP^+^) tumor cells at a 1:4 ratio in serum-free medium at 37°C for 4 hours in low-attachment 96-well tissue culture plates (Corning, USA). The cells were harvested, and primary macrophages were identified by flow cytometry using anti-F4/80 or anti-CD14 antibody (eBioscience, USA). Then, 7-AAD (eBioscience, USA) was added to exclude dead cells in some experiments. Phagocytosis rate was determined as the percentage of CFSE^+^ or GFP^+^ macrophages.

### Tumor models and treatments

C57BL/6 mice were subcutaneously (s.c.) injected with 1×10^6^ MC38 cells or 2×10^5^ B16-OVA into the flank, and BALB/c mice were s.c. injected with 2×10^5^ CT26 cells into the flank. Tumor volumes were measured every other day by length (a), width (b) and height (c), and calculated as tumor volume=abc/2. Tumors were grown for approximately a week, the mice were then injected s.c. with 2 mg/kg pep-20 or normal saline as the negative control at the peritumoral site every day. Treatment was continued for 2 weeks, then the mice were euthanized, tumors, spleens and draining lymph nodes were dissected. For macrophages depletion, C57BL/6 mice were intraperitoneally (i.p.) injected with 150 µL clodronate liposome or control liposome (FormuMax Scientific, USA) on day 6, and every 4 days after injection of 1×10^6^ MC38 cells until finishing the experiment. The efficiency of macrophages depletion was determined by flow cytometry analysis of the CD45^+^ CD11b^+^ F4/80^+^ cells. For pep-20-D12 treatment, 1×10^6^ MC38 cells were injected s.c. into the flank of mice, and 2 mg/kg pep-20-D12 every day or 400 µg anti-mouse CD47 antibody (miap301, Bio-XCell, USA) every 3 days for a total of five times as the positive control was injected i.p. into the mice, with normal saline and ratIg as the negative controls. For radiotherapy (RT), 1×10^6^ MC38 cells were injected s.c. into the flanks of mice, tumors were allowed to grow to reach 80 to 100 mm^3^ before being treated with RT. Tumors locally received one 20 Gy dose IR and then 2 mg/kg pep-20-D12 was injected i.p. into the mice every day for 2 weeks.

For CD8^+^ T cells detection in tumors, single cell suspensions from tumor tissues were prepared by gentle mechanical disruption, the tumor masses were then digested with collagenase IV (Invitrogen, USA) and Dnase I (Sigma, USA) for 30 min at 37°C, and cells were passed through a 70 µm nylon cell strainer. Tumor cells were stained with anti-mouse CD45, anti-mouse CD3, anti-mouse CD8α or isotype control, and then analyzed by flow cytometry.

For intracellular cytokine staining assay, tumor-infiltrating lymphocytes (TILs) were purified using Ficoll-gradient centrifugation (GE, USA). Single cell suspensions from TILs, draining lymph nodes or spleens were re-stimulated with 20 ng/mL phorbol 12-myristate 13-acetate (PMA, Sigma, USA) and 1 µM ionomycin (Sigma, USA) for 4 hours to CT26 and MC38 tumor-bearing mice, or 10 µg/mL OVA_257-264_ peptide for 6 hours to B16-OVA tumor-bearing mice, in the presence of protein transport inhibitor cocktail (eBioscience, USA). Interferon (IFN)-γ production from CD8^+^ T cells was detected with anti-mouse CD3, anti-mouse CD8α, and anti-mouse IFN-γ or isotype control, and then analyzed by flow cytometry.

For ELISA assay, spleen or draining lymph node cells were re-stimulated with 0.5 µg/mL anti-CD3 and 0.5 µg/mL anti-CD28 antibodies (eBioscience, USA) for 3 days to CT26 and MC38 tumor-bearing mice, or 10 µg/mL OVA_257-264_ peptide for 5 days to B16-OVA tumor-bearing mice. Cellular supernatant IFN-γ secretion was measured by using an ELISA kit (eBioscience, USA).

### Statistical analysis

Statistical analysis were performed with unpaired Student’s t-test for analyzing differences between groups. The Kaplan-Meier curve and the log-rank test were used for analyzing overall survival rate of the mice. Data were represented as means±SEM unless otherwise indicated. *p<0.05, **p<0.01 and ***p<0.001 were considered statistically significant.

## Results

### CD47 is significantly overexpressed in tumors

The CD47 expression in different tumor tissues from The Cancer Genome Atlas (TCGA) and Genotype-Tissue Expression (GTEx) data sets was evaluated. It was found that CD47 expression was markedly higher in various kinds of tumor tissues compared with normal counterparts (online supplemental figure S1A). Consistently, CD47 was expressed at high levels on various solid and hematologic tumor cell lines shown by flow cytometry analysis (online supplemental figure S1B). As previous studies have shown,^21^ the high expression of CD47 in tumors suggested the potential of targeting CD47 for antitumor immunotherapy.

10.1136/jitc-2020-000905.supp1 Supplementary data

### Bio-panning and identification of peptides binding CD47 via phage display strategy

To identify the peptides binding to human CD47, the solution-phase biopanning was performed with affinity beads capture strategy, which improved accessibility of the protein-binding site according to the standard protocol of the Ph.D.-12 phage display peptide library using human CD47-IgV-Domain protein as the target.^22^ Phages that are not specifically binding with CD47 can be avoided by employing a negative selection strategy. After five rounds of bio-panning, phages of enrichment were obtained and phage plaques were randomly selected for DNA sequencing. Subsequently, the sequences were aligned using Clustal Omega,^23^ and several consensus sequences were apparently different, such as ‘YKEHYLY’ and ‘TXSNY’ (online supplemental figure S2). This indicated that the binding regions of these phages to CD47 might not be the same cluster. Therefore, in order to reduce the ‘off-rate’, peptides with different common sequences were accepted. Based on the selection strategy, 12 candidate peptides were further screened to identify which could bind to human CD47 (online supplemental table S1).

10.1136/jitc-2020-000905.supp2 Supplementary data

### Pep-20 binds to CD47 and blocks the CD47/SIRPα interaction

**Figure 1:**
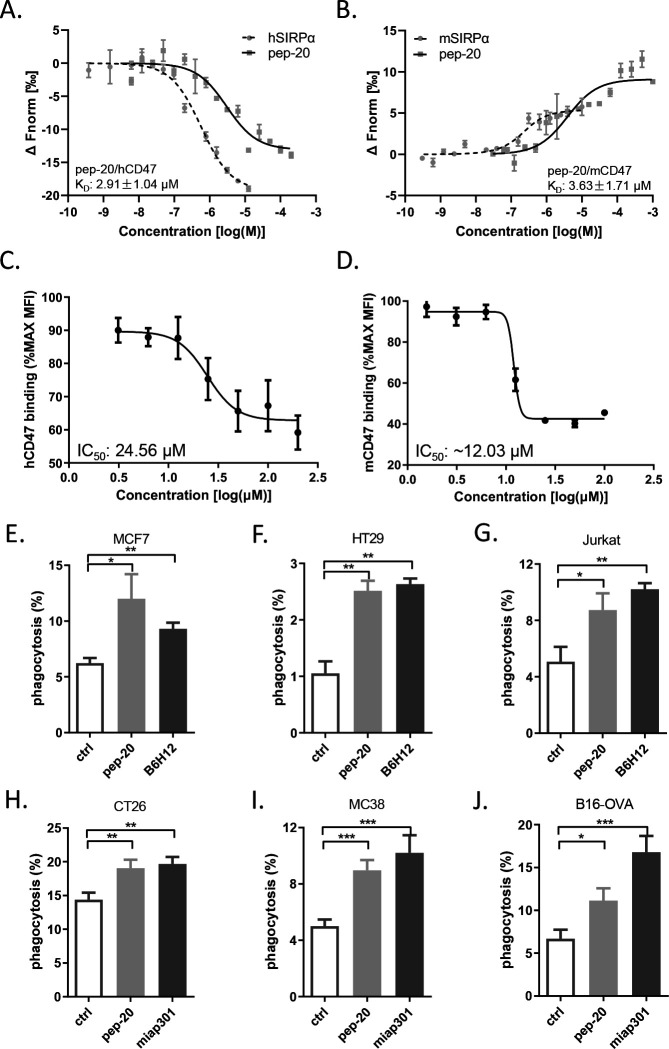
Pep-20 binds to CD47 and blocks the CD47/SIRPα interaction. (A, B) Dose response curves of pep-20 binding to CD47. Binding assay of pep-20 to human (A) or mouse (B) CD47-IgV-Domain protein was examined by the MST. The human or mouse SIRPα protein served as positive control. (C, D) Dose response curves of pep-20 interfering CD47/SIRPα interaction. Flow cytometry analysis of human (C) or mouse (D) CD47-IgV-Domain-hIg fusion protein binding to CHO stably expressing human or mouse SIRPα cells in the presence of pep-20 with varying gradient concentrations. The data represented as the mean fluorescence intensity normalized to the maximum binding. (E to J) Phagocytosis assays were performed by co-culture of tumor cells with corresponding macrophages at a 1∶4 ratio in serum-free medium at 37℃ for 4 hours in low-attachment 96-well plates. GFP^+^ MCF7 cells (E), GFP^+^ HT29 cells (F) and GFP^+^ Jurkat cells (G) were incubated with human peripheral blood-derived macrophages in the presence of 100 µM pep-20 or 20 µg/mL anti-human CD47 antibody (B6H12). CFSE^+^ CT26 cells (H), GFP^+^ MC38 cells (I) and GFP^+^ B16-OVA cells (J) were incubated with mouse bone marrow-derived macrophages in the presence of 100 µM pep-20 or 20 µg/mL anti-mouse CD47 antibody (miap301). Phosphate-buffered saline was negative control in all assays. CFSE^+^ or GFP^+^ macrophages were detected by flow cytometry. Data are represented as means±SEM. Statistical significance was determined by unpaired Student’s t-test. *p<0.05; **p<0.01; ***p<0.001. CHO, Chinese hamster ovary; CSFE, carboxyfluorescein succinimidyl ester; GFP, green fluorescent protein; MST, microscale thermophoresis.

To assess the affinity of these candidate peptides toward human CD47, the preliminary binding assay was performed. Several peptides were confirmed to bind human CD47 with a relatively strong affinity comparable to the native CD47/SIRPα interaction, such as pep-3 and pep-20. In addition, due to the presence of consensus sequences, it can be hypothesized that the peptides pep-5, pep-9 and pep-19 may have higher affinities. But due to the poor solubility, it’s not available to use the higher titrating concentrations to determine the saturation binding concentration (online supplemental table S1). Subsequently, pep-20 was examined to bind to human and mouse CD47 with the K_D_ values of 2.91±1.04 µM and 3.63±1.71 µM, respectively (figure 1A, B).

To evaluate the ability of the peptide candidates to block the CD47/SIRPα interaction, the blocking assay was performed in vitro. Three peptides (pep-15, pep-16 and pep-20) could block the interaction of CD47/SIRPα (online supplemental figure S3), among which pep-20 exhibited the greatest dose-dependent effect with an IC_50_ of 24.56 µM (figure 1C). The anti-CD47 antibody (B6H12) served as a positive control with an IC_50_ of 6.94 µg/mL (online supplemental figure S4). The same experiment was also performed in a mouse blocking system, and the blockade of pep-20 to mouse CD47/SIRPα interaction was determined with an IC_50_ of 12.03 µM approximately (figure 1D). These results clearly demonstrated that pep-20 could bind to CD47 and block the interaction of CD47/SIRPα.

### Pep-20 enhances macrophages-mediated phagocytosis of tumor cells

Previous studies showed that phagocytosis of macrophages was the major mechanism of CD47 targeted therapies.^8^ Thus, the in vitro capability of pep-20 to facilitate macrophages-mediated phagocytosis of tumor cells was investigated. Three different human tumor cell lines of MCF7, HT29 and Jurkat were offered to macrophages as target cells, and the results showed that pep-20 significantly increased macrophages-mediated phagocytosis of tumor cells, which was slightly inferior to the anti-CD47 antibody (B6H12) positive control (figure 1E–G). Subsequently, whether the mouse macrophages possessed efficacy of phagocytosis towards murine tumor cells in the presence of pep-20 was also investigated. As a result, similar phagocytosis occurrences were observed from CT26, MC38 and B16-OVA tumor cells treated with pep-20 (figure 1H–J). To confirm the specificity of pep-20 targeting CD47, the CD47 knockdown HT29 and MC38 tumor cells were established. CD47 knockdown impairs the efficacy of pep-20 to induce macrophage-mediated phagocytosis of tumor cells (online supplemental figure S5). These results demonstrated that pep-20 might have potential therapeutic activity in tumor-bearing mice.

### Pep-20 reduces the tyrosine phosphorylation of SIRPα via CD47 engagement

To further verify the ability of pep-20 to block CD47/SIRPα interaction, the SIRPα tyrosine residues phosphorylation with pep-20 intervention was evaluated.^24^ As a negative regulation signal of CD47/SIRPα axis, the ligation of SIRPα by CD47 could lead to tyrosine phosphorylation of immunoreceptor tyrosine-based inhibitory motifs (ITIMs) in its cytoplasmic domain.^25^ For the validity of the experimental results, SIRPα expression on several murine tumor cells was evaluated and it was found that SIRPα was not detected on CT26 cells. Following incubation of mouse bone marrow-derived macrophages with CT26 cells, it was found that pep-20 could reduce the tyrosine residues phosphorylation of SIRPα, which was consistent with that of anti-CD47 antibody (miap301) (online supplemental figure S6). The results clearly demonstrated that pep-20 could directly block the CD47/SIRPα interaction.

### Pep-20 inhibits tumor growth and activates antitumor T-cell immune response

**Figure 2:**
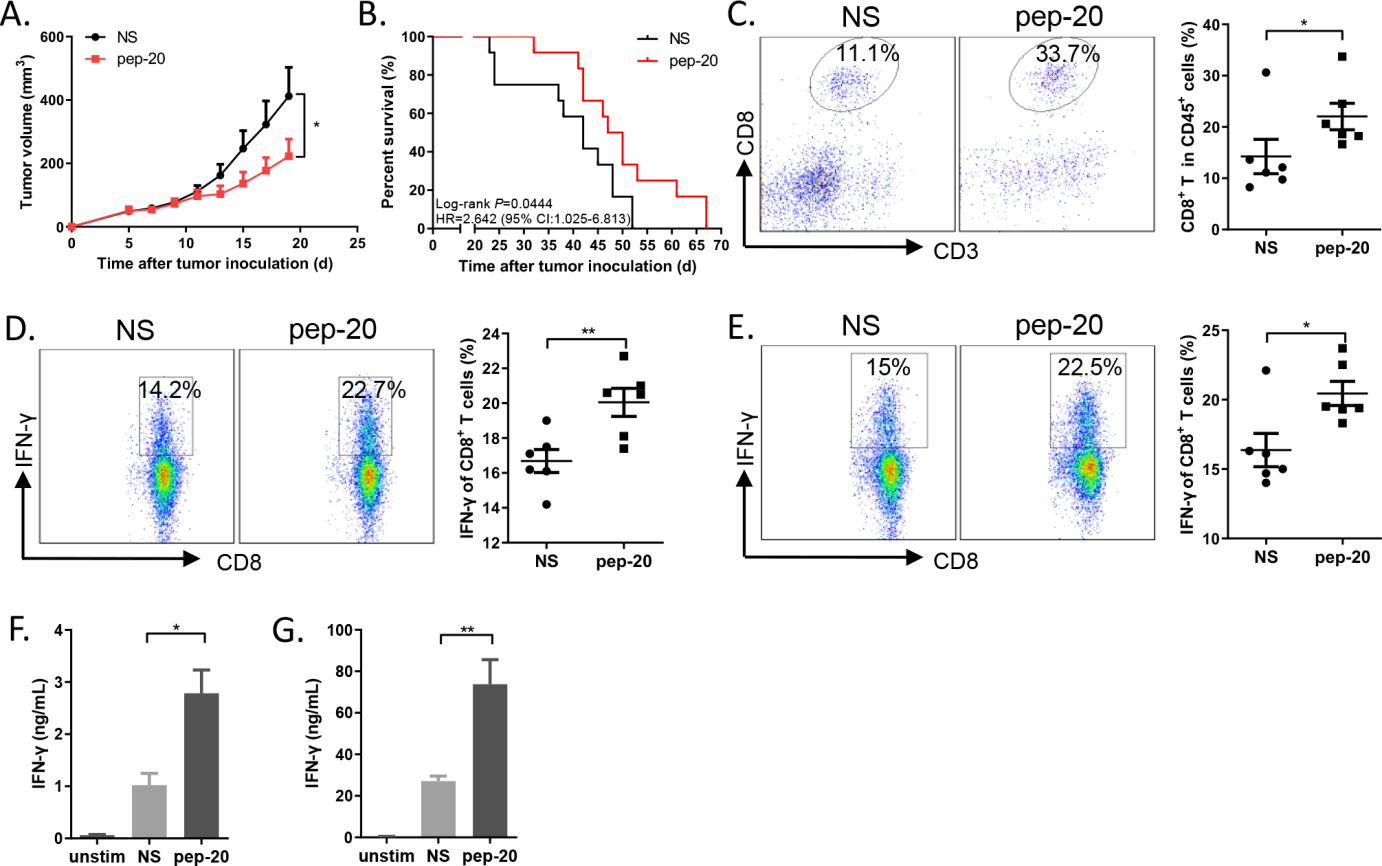
Pep-20 inhibits tumor growth and activates antitumor T-cell immune response in MC38 tumor-bearing mice. C57BL/6 mice were transplanted with 1×10^6^ MC38 cells on the right flank, until the tumor volume reached 50 mm^3^. (A) Mice were treated s.c. with 2 mg/kg of pep-20 or normal saline as the negative control at the peritumoral site every day for 2 weeks (n=8). (B) Mice were treated with 2 mg/kg of pep-20 for 4 weeks and overall survival was monitored (n=12). (C) Tumors were detected for the percentage of tumor-infiltrating CD8^+^ T cells in total CD45^+^ cells (n=6). (D, E) Cells from draining lymph nodes (D) or spleen (E) were obtained and stimulated with 20 ng/mL of PMA and 1 µM ionomycin-containing protein transport inhibitor cocktail for 4 hours. Frequencies of IFN-γ expressing CD8^+^ T cells were detected by flow cytometry (n=6). (F, G) Draining lymph nodes (F) and spleen (G) were obtained and stimulated with 0.5 µg/mL of anti-CD3 and 0.5 µg/mL of anti-CD28 antibodies for 3 days. Cellular supernatant from draining lymph nodes or spleen for IFN-γ secretion was measured with ELISA assay (n=4 to 5). Data are represented as means±SEM. Statistical significance was determined by unpaired Student’s t-test. *p<0.05; **p<0.01. Kaplan-Meier survival curves were evaluated by log-rank analysis. IFN, interferon; PMA, phorbol 12-myristate 13-acetate.

The antitumor efficacy of pep-20 in tumor-bearing mice was subsequently investigated. MC38 cells were engrafted s.c. on the flank of mice. Tumor growth was significantly suppressed by pep-20 treatment (2 mg/kg), and mice displayed prolonged overall survival compared with the negative control of normal saline (figure 2A, B). To confirm these results, the antitumor efficacy of pep-20 was also evaluated using similar treatment program in CT26 tumor-bearing mice. The delay of tumor growth and prolonged overall survival were also observed (online supplemental figure S7A, B).

To determine whether pep-20 treatment engages antitumor immune response, the cells from the tumor tissues, tumor-draining lymph nodes and spleen were obtained after treatment. CD47 blockade with pep-20 remarkably increased the intratumoral CD8^+^ T cell population (figure 2C). In addition, IFN-γ expressing CD8^+^ T cells from tumor-draining lymph nodes and spleen were significantly increased in pep-20 treated MC38 tumor-bearing mice (figure 2D–G). These results were also confirmed in CT26 model (online supplemental figure S7C-G).

**Figure 3:**
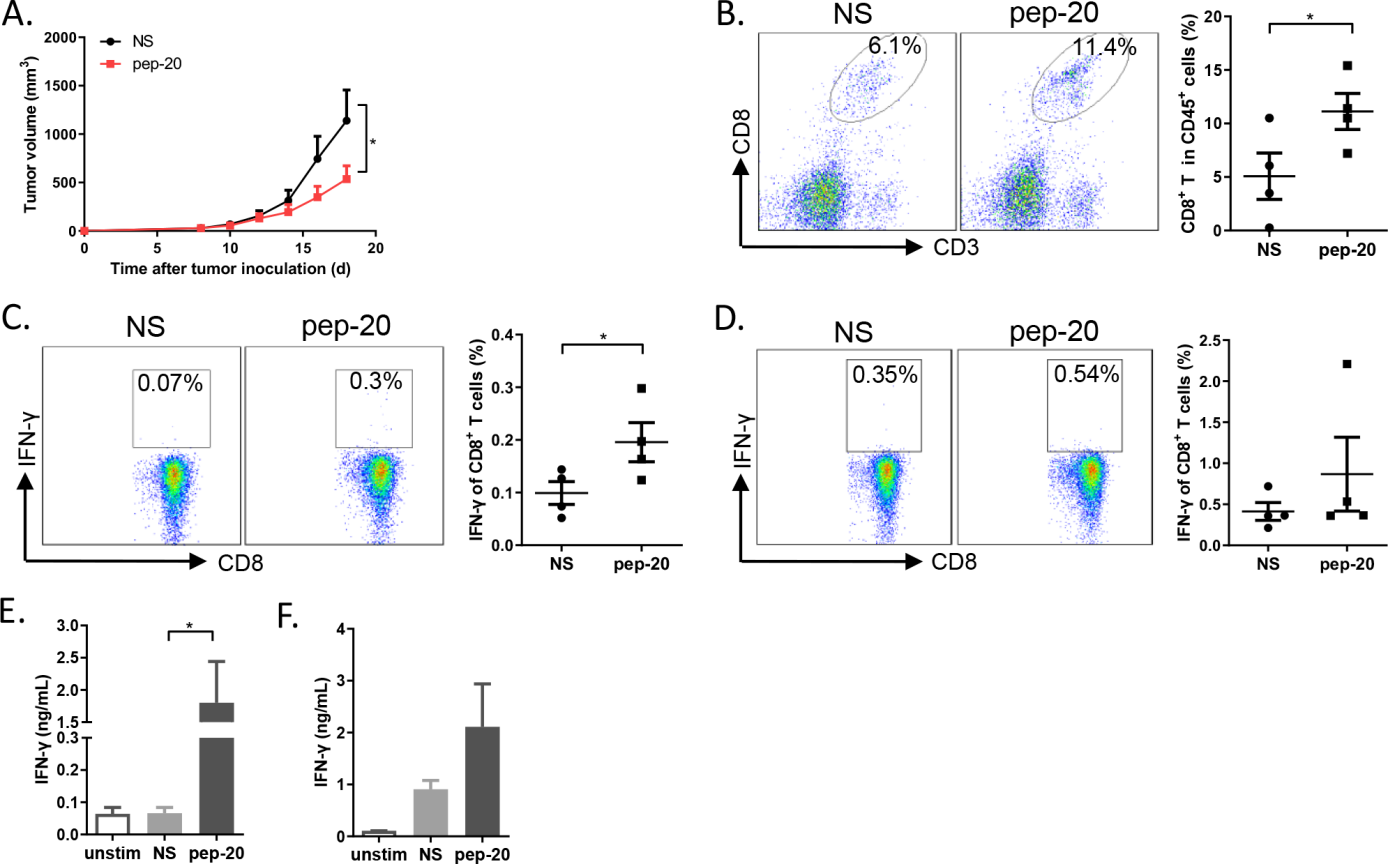
Pep-20 inhibits tumor growth and activates specific antitumor T-cell immune response in B16-OVA tumor-bearing mice. C57BL/6 mice were injected with 2×10^5^ B16-OVA cells on the right flank until the tumor volume reached 30 mm^3^. (A) Mice were treated s.c. with 2 mg/kg of pep-20 or normal saline as the negative control at the peritumoral site every day for 2 weeks (n=5 to 6). (B) Tumors were detected for the percentage of tumor-infiltrating CD8^+^ T cells in total CD45^+^ cells (n=4). (C, D) Cells from draining lymph nodes (C) or spleens (D) were obtained and stimulated with 10 µg/mL of OVA_257-264_ peptide containing protein transport inhibitor cocktail for 6 hours. Frequencies of IFN-γ expressing CD8^+^ T cells were detected by flow cytometry (n=4). (E, F) Cells from draining lymph nodes (E) or spleens (F) were obtained and stimulated with 10 µg/mL of OVA_257-264_ peptide for 5 days. Cellular supernatant from draining lymph nodes and spleen for IFN-γ secretion was measured with ELISA assay (n=4). Data are represented as means±SEM. Statistical significance was determined by unpaired Student’s t-test. *p<0.05; **p<0.01. IFN, interferon.

Based on these results, we wondered whether pep-20 could enhance tumor antigen specific T-cell immune response. To verify this hypothesis, B16-OVA tumor-bearing mice was established. Consistently, pep-20 treatment could significantly suppress tumor growth (figure 3A). The cells from the tumor-draining lymph nodes and spleen were stimulated with OVA_257-264_ peptide ex vivo after treatment. Pep-20 significantly increased the CD8^+^ T cell population in tumor tissue (figure 3B). Identically, IFN-γ expressing CD8^+^ T cells (stimulated by antigenic peptide OVA_257-264_) in major peripheral lymphoid organ from tumor-bearing mice were also increased after pep-20 treatment, especially in draining lymph nodes (figure 3C–F). Collectively, these results demonstrated that blockade of CD47 with pep-20 could delay the tumor growth and induce antigen-specific T-cell immune response.

### Pep-20 enhances the activity of CD8^+^ T cells via macrophages

Based on the results described above, it can be presumed that macrophages could mobilize the T-cell response after phagocytosis of tumor cells by pep-20. To further verify the effects of pep-20-mediated phagocytosis by macrophages to CD8^+^ T-cell response, the proliferation of OT-I T cells was evaluated in vitro. As predicted, macrophages could significantly enhance the activity of CD8^+^ T cells after engulfing B16-OVA cells in the presence of pep-20, which was consistent with the anti-CD47 antibody (miap301) (online supplemental figure S8A, B). In addition, more secretion of IFN-γ from CD8^+^ T cells in the culture supernatants was detected in pep-20-treated group (online supplemental figure S8C). These results demonstrated that the phagocytosis of macrophages enhanced by pep-20 was able to stimulate antitumor CD8^+^ T-cell immune response.

### Depletion of macrophages impairs the antitumor efficacy of pep-20 in vivo

**Figure 4:**
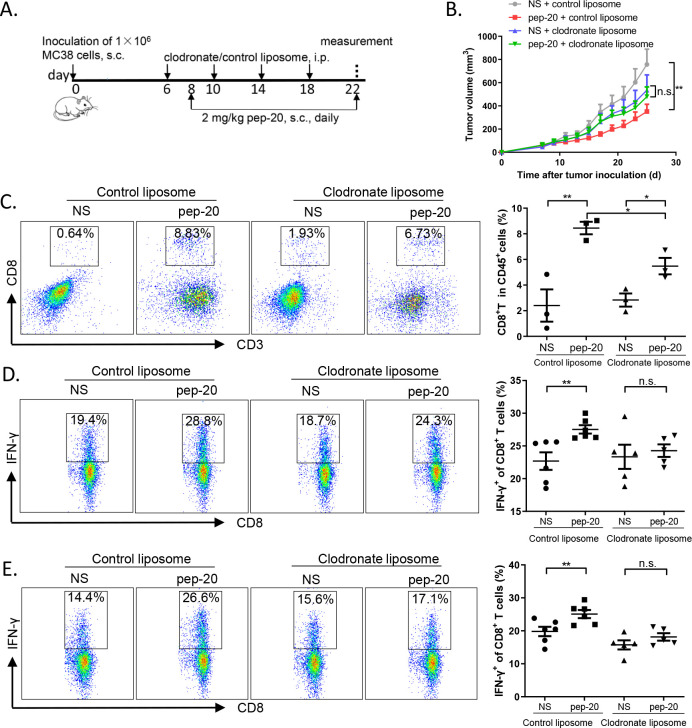
Depletion of macrophages impairs the antitumor efficacy of pep-20 in vivo. C57BL/6 mice were transplanted with 1×10^6^ MC38 cells on the right flank, until the tumor volume reached 50 mm^3^. (A, B) Mice were treated s.c. with 2 mg/kg of pep-20 or normal saline as the negative control at the peritumoral site every day, starting on day 8 for 2 weeks and 150 µL clodronate liposomes or control liposomes were injected i.p. on days 6, 10, 14 and 18. (n=5 to 6). (C) Tumors were detected for the percentage of tumor-infiltrating CD8^+^ T cells in total CD45^+^ cells (n=3). (D, E) Cells from draining lymph nodes (D) and spleens (E) were obtained and stimulated with 20 ng/mL of PMA and 1 µM ionomycin-containing protein transport inhibitor cocktail for 4 hours. Frequencies of IFN-γ-expressing CD8^+^ T cells were detected by flow cytometry (n=5 to 6). Data are represented as means±SEM. Statistical significance was determined by unpaired Student’s t-test. *p<0.05, **p<0.01. IFN, interferon; i.p. intraperitoneally; n.s., no significance; PMA, phorbol 12-myristate 13-acetate; s.c., subcutaneously.

To further demonstrate whether macrophages directly contribute to the antitumor effects of pep-20, macrophages were depleted with clodronate liposome before injection of pep-20 in MC38 tumor-bearing mice.^26^ Prior to the experiment, the macrophages were confirmed to be removed effectively in peripheral blood and spleen compared with control liposome (online supplemental figure S9). The detailed description of the treatment program is presented in figure 4A. By monitoring tumor sizes of MC38-bearing mice, no obvious difference was observed between pep-20 and normal saline groups in the presence of clodronate liposomes. In contrast, tumor growth was markedly suppressed by pep-20 compared with normal saline group in the presence of control liposomes (figure 4B). Overall, these findings indicated that macrophage depletion was able to impair the therapeutic efficacy of pep-20.

To further verify whether depletion of macrophages could attenuate the effects of pep-20 on antitumor immune response, the activity of CD8^+^ T cells after clodronate liposomes treatment was studied. When the macrophages were depleted, a significant reduction in the percentages of intratumoral CD8^+^ T cells was observed with pep-20 treatment, compared with the mice without macrophages depletion (figure 4C). Additionally, the IFN-γ-expressing CD8^+^ T cells from the draining lymph nodes and spleen following stimulation with PMA and ionomycin ex vivo were examined. The secretion of IFN-γ from CD8^+^ T cells showed no significant differences with or without pep-20 treatment after macrophages depletion despite the increase of CD8^+^ T cells population in the tumor tissue, indicating the critical role of macrophages in mediating the antigen cross-presentation and thus activating the CD8^+^ T cells (figure 4C–E). Therefore, these results indicated that the antitumor effects of pep-20 were dependent on CD8^+^ T-cell activation mediated by macrophages.

### Pep-20 displays no significant toxicity in mice

Previous studies have demonstrated that CD47 is expressed on a variety of cells, including hematopoietic cells.^6^ Therefore, the blood toxicity caused by CD47/SIRPα blockade has received more attention. To preliminarily assess the toxicity of pep-20 in vivo, C57BL/6 naïve mice were injected s.c. with pep-20 at a dose of 2 mg/kg daily over 14 days. Blood samples were collected, and hematological parameters were analyzed either before or after pep-20 treatment. It was found that the red blood cell count and hemoglobin level had no significant differences (online supplemental figure S10A, B). Subsequently, the organ coefficients and hepatic damage analysis also revealed no significant differences after pep-20 treatment (online supplemental table S2 and S3). Consistently, histopathology analysis illustrated no abnormalities by H&E staining of tissue sections from major organs (online supplemental figure S10C). Collectively, the results demonstrated that pep-20 could not induce anemia and other obvious side effects at a dose of 2 mg/kg in mice.

### Prediction of docking model of pep-20 to CD47

To determine the binding detail of pep-20 to CD47, the 3D conformation of pep-20 was predicted by PEP-FOLD3, as shown by using the MOE software (online supplemental figure S11A). Next, the possible interaction of pep-20 with human CD47 protein (PDB ID: 2JJS)^27^ was predicted by ZDOCK. In order to confirm the binding model, pep-20-derived alanine substitution analogs were tested by cell-based blocking assay. As shown in online supplemental figure S11B, the predicted W2, W6, Y9 and W10 residues may be the major binding sites. Subsequently, a possible binding pattern complex 796 was obtained by correlation analysis of the top 1000 predictions (online supplemental figure S11C), and the docking model and interaction residues of CD47/pep-20 were displayed (online supplemental figure S11A). It was found that the binding sites of CD47/pep-20 were located exactly within the CD47/SIRPα interaction interface (online supplemental figure S11D). These findings revealed the docking model of pep-20, which lays down a potential platform to generate more potent derivates in the future.

### Pep-20-D12 blocks the CD47/SIRPα interaction and enhances proteolysis stability compared with parent pep-20

To develop a more stable peptide which could be administered systemically, modification of pep-20 with D-amino acid substitution was attempted. According to the docking model on the interaction of pep-20 and CD47, and to ensure the blockading effect and hydrolysis stability, both N-terminal and C-terminal residues were systematically substituted by up to three D-amino acids (online supplemental table S4). The blocking assay was performed as mentioned above. The results showed that pep-20-D12, with three D-amino acid residues substituted both in N-terminal and C-terminal of the parent pep-20, could retain the blockade activity toward human and mouse CD47/SIRPα (online supplemental figure S12A, B), indicating that the N-terminal and C-terminal residues of pep-20 could be substituted without significant functional decrease. Subsequently, the enzymatic degradation stability was examined in 10% human serum at 37°C. Different from parent pep-20, pep-20-D12 exhibited potent proteolytic resistance, which retained the same initial concentration for up to 36 hours (online supplemental figure S12C, D). In addition, pep-20-D12 showed more prolonged half-life (T_1/2_) in vivo with the intravenous elimination T_1/2_ of 6.36 hours and C_max_ of 54.45 µg/mL (40 mg/kg) in C57BL/6 mice than pep-20 with T_1/2_ of 0.59 hours and C_max_ of 33.60 µg/mL (online supplemental figure S12E, F). These findings suggested that the stability of pep-20 could be greatly improved by terminal D-amino acid substitution.

### Pep-20-D12 inhibits the growth of tumors and activates antitumor T-cell immune response via systemic administration

In view of the evidence that pep-20-D12 has better proteolytic stability than parent pep-20, the antitumor efficacy and immune stimulatory activity of both peptides were performed in MC38-bearing mice via systemic treatment. Pep-20-D12 slowed the tumor progression significantly, whereas pep-20 had a slight impact on tumor growth (online supplemental figure S13A). Similarly, pep-20-D12 enhanced the infiltration of tumor-specific T cells and antitumor immunity (online supplemental figure S13B-D).

**Figure 5:**
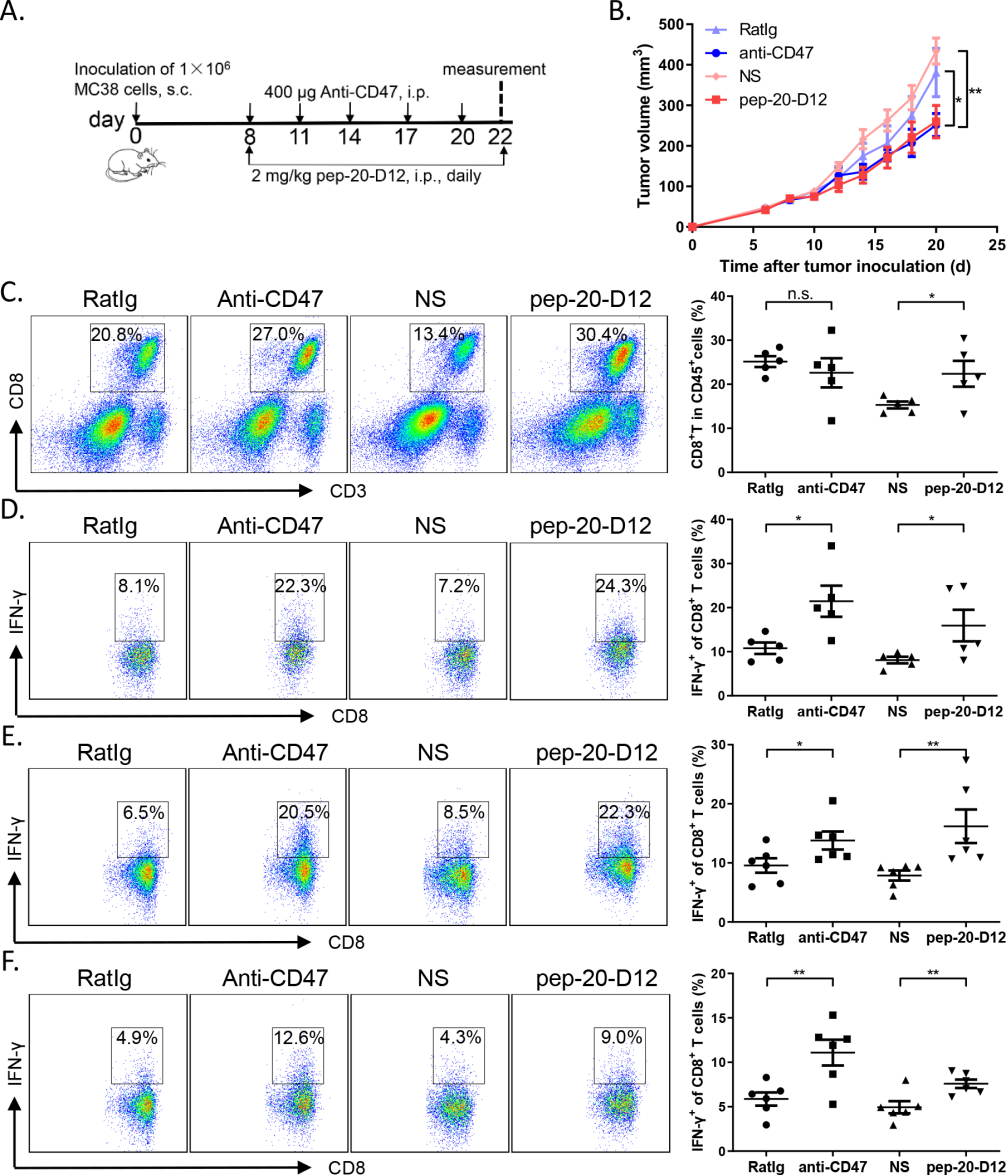
Pep-20-D12 inhibits the growth of tumors and activates antitumor T-cell immune response via systemic administration. C57BL/6 mice were transplanted with 1×10^6^ MC38 cells on the right flank, until the tumor volume reached 50 mm^3^. (A, B) Mice were treated i.p. with 2 mg/kg of pep-20-D12 every day for 2 weeks or 400 µg of anti-mouse CD47 antibody (miap301) every 3 days for a total of five times as the positive control, and normal saline and RatIg as the negative controls (n=8). (C) Tumors were detected for the percentage of tumor-infiltrating CD8^+^ T cells in total CD45^+^ cells (n=5). (D to F) Cells from tumor-infiltrating lymphocytes (D), draining lymph nodes (E) or spleens (F) were obtained and stimulated with 20 ng/mL of PMA and 1 µM ionomycin-containing protein transport inhibitor cocktail for 4 hours. IFN-γ-expressing CD8^+^ T cells were detected by flow cytometry. (n=5). Data are represented as means±SEM. Statistical significance was determined by unpaired Student’s t-test. *p<0.05; **p<0.01. IFN, interferon; i.p. intraperitoneally; n.s., no significance; PMA, phorbol12-myristate 13-acetate; s.c., subcutaneously.

Consistently, the tumor growth inhibition efficacy by systemic treatment of pep-20-D12 was similar as the anti-CD47 antibody (figure 5A, B). To examine the effects of pep-20-D12 treatment on antitumor T-cell immune response, CD8^+^ T cells from tumor tissues, tumor-draining lymph nodes and spleen were analyzed. Blockade of CD47 with systemically pep-20-D12 treatment also remarkably increased the CD8^+^ T cell population in tumor tissues (figure 5C). Furthermore, IFN-γ production by CD8^+^ T cells from TILs, tumor-draining lymph nodes and spleen was also significantly increased, especially in TILs and draining lymph nodes (figure 5D–F). More importantly, pep-20-D12 showed less blood toxicity compared with anti-CD47 antibody by evaluating the hematological parameters of mice after treatment (online supplemental figure S14A, B). These results indicated that pep-20-D12 maximally extended antitumor effects with systemic administration.

### Combination of pep-20-D12 with irradiation synergistically inhibits tumor growth

**Figure 6:**
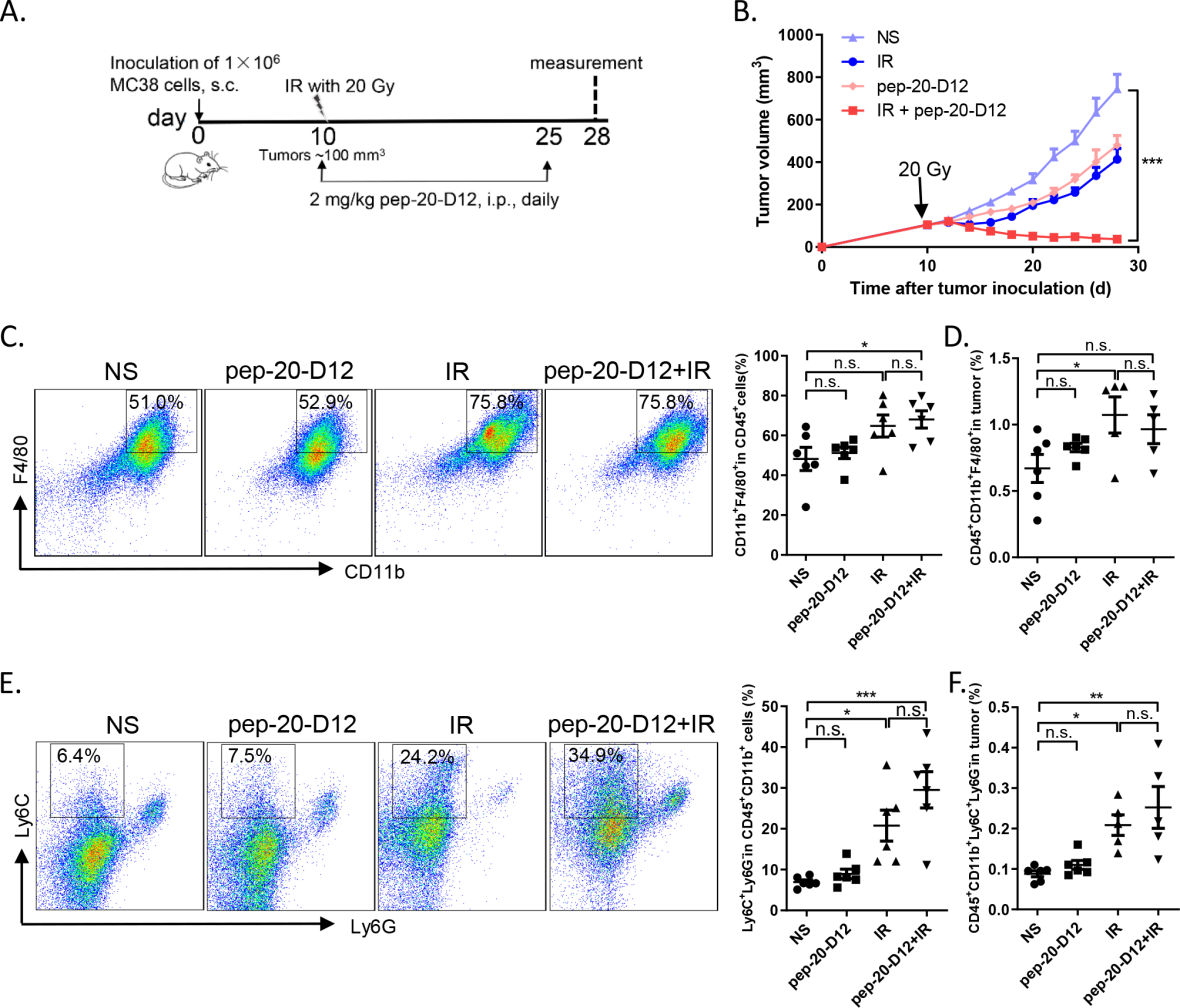
Combination of pep-20-D12 treatment with IR synergistically delays tumor growth. C57BL/6 mice were transplanted with 1×10^6^ MC38 cells on the right flank, and tumors were allowed to grow to ~100 mm^3^ before treatment with IR. (A, B) Tumors locally received one 20 Gy dose IR, and 2 mg/kg of pep-20-D12 was daily i.p. injected from the same day of IR for 2 weeks. Tumor growth was monitored after IR (n=7). (C, F) Infiltrating immune cell subsets in tumors were analyzed after treatment. The percentages of intratumoral macrophages (CD45^+^CD11b^+^F480^+^) (C, D) and monocyte-derived MDSCs (CD45^+^CD11b^+^Ly6C^+^Ly6G^-^) (E, F) in total tumor tissues were evaluated by ﬂow cytometry (n=5 to 6). Data are represented as means±SEM, Statistical significance was determined by one way analysis of variance. *p<0.05; **p<0.01; ***p<0.001. i.p.intraperitoneally; IR, irradiation; MDSC, myeloid-derived suppressor cells; n.s., no significance; s.c., subcutaneously.

It was reported that the proportion of monocytic myeloid-derived suppressor cells (M-MDSCs) in tumor tissues increased significantly after local IR in mice.^28^ Considering that these cells could be differentiated into macrophages subsequently,^29 30^ we attempted to evaluate whether combined pep-20-D12 treatment with IR could be more effective to inhibit tumor growth than either treatment alone. MC38 tumors locally received IR once (20 Gy) and were then treated by injection i.p. of pep-20-D12 daily for 2 weeks. Surprisingly, a combination of pep-20-D12 treatment with IR showed a significant delay or even complete regression in tumor growth (figure 6A, B). The mechanism study indicated that the proportion of tumor infiltrating macrophages and monocyte-derived MDSCs was significantly increased on day 3 and the end of treatment by IR (online supplemental figure S15A, B, figure 6C-F). More importantly, pep-20-D12 could promote the tumor infiltrating monocyte-derived MDSCs-mediated phagocytosis of tumor cells (online supplemental figure S15C). These results demonstrated that the combination of CD47 blockade pep-20-D12 peptide with IR could be a very promising strategy to synergistically eradicate the tumor.

## Discussion

CD47, as a ubiquitous cell-surface inhibitory receptor, interacts with SIRPα on phagocytic cells to deliver inhibitory signals, thus inhibiting the phagocytosis and evading immune surveillance. Recent preclinical or clinical studies suggested that the strategies of blocking anti-phagocytic CD47/SIRPα interaction to enable macrophages phagocytosis of tumor cells showed promise in cancer therapy.^31 32^Here, our results provided the evidence about the overexpression of CD47 in human colorectal tumor tissues compared with the matched normal tissues, and the increased CD47 expression negatively correlated with overall survival, which indicated poor clinical outcomes. Based on these results, a CD47-targeting peptide, pep-20, was identified to specifically bind to human CD47-IgV-Domain and block the CD47/SIRPα interaction. Western blotting results revealed that pep-20 directly disturbed the CD47/SIRPα interaction by inhibiting SIRPα tyrosine phosphorylation of ITIMs in cytoplasmic domain.

Surprisingly, pep-20 could bind to human or mouse CD47 in a dose-dependent manner as well as elicit blockade effects. The sequence identity of human and mouse CD47 is 76%. It was also found the sequences of natural receptor-ligand interaction regions determined by crystal structure analysis exihibited a higher overlap.^27^ The binding regions in which pep-20 interact with CD47 might be highly consistent between human and mouse. Blocking assays of pep-20-derived alanine substitution analog peptides were performed to verify the major contact residues. The predicted binding sites of pep-20 to CD47 were presented in the interaction regions of natural CD47/SIRPα obtained by crystal structure analysis, indicating pep-20 could directly block their interaction. Above all, the docking model can be exploited for performing various studies, such as structural simulation and molecular docking, which could provide the binding detail of peptides interact with receptors, and thus provide more relevant information for the optimization of peptides in the future.^33^

Moreover, the results indicated that pep-20 could act as a blocker to enable engulfment of tumor cells by macrophages in vitro and in vivo. Blockade effects of disrupting the CD47/SIRPα interaction elicit direct macrophages-mediated phagocytosis rather than antibody-dependent cell-mediated cytotoxicity (ADCC) or other mechanisms to disrupting the interaction. Due to the ubiquitous expression of CD47 on various cells, including erythrocytes and platelets, in addition to high expression on tumor cells, the side effects of hematological toxicity caused by non-specific clearance has been a major drawback for CD47 targeting cancer therapy, which may restrict the clinical applications of CD47 blockade. Therefore, the agents targeting CD47 have shown some disadvantages, including the trouble in determining the administered doses to avoid the side effects as well as the low bioavailability caused by disordered binding and clearance of erythrocytes in vivo.^34 35^ To overcome this, many strategies have been attempted to simply block anti-phagocytic signals using single-chain variable fragment, the IgG4 isotype Fc region instead of IgG1 or other molecules which are not dependent on the ADCC activity.^36 37^ In the present study, C57BL/6 mice received pep-20 and its D-amino acid derivate treatment showed no significant adverse effects such as anemia, liver and kidney damage. Thus, it can be speculated that the peptides absenting functional IgG1 Fc regions are not able to trigger FcR-mediated ADCC and complement-dependent cytotoxicity effects.

Peptide treatments can activate macrophages-mediated phagocytosis through direct blockade effects which weaken the off-target clearance of erythrocytes. Moreover, Advani *et al* proposed that CD47 blockade with a low dose at the beginning, followed by a higher grade maintenance dose, minimized the side effects of anemia.^32^ Our work was partly consistent with this strategy. Compared with antibodies and fusion proteins, the T_1/2_ of peptides is relatively short, which may weaken the therapeutic effect to some extent. In return, adverse effects could be managed timely and effectively for clinical treatments. Furthermore, administering a large dose of inhibitors in a short period may break the balance of pro-phagocytic and anti-phagocytic signals which are partly regulated by cellular homeostasis, even normal cells are not subjected to phagocytosis because of lack of pro-phagocytic signaling.^38^ In response to erythrocyte decline, peptide treatment may be a relatively milder strategy that enables maintaining relative homeostasis or returning to a stable state after a short period of disequilibrium via a compensatory effect. While macrophages are the main effector cells of CD47/SIRPα pathway from current studies, it is undeniable that the presence of Fc receptor (FcR)-mediated ADCC and antibody-dependent cellular phagocytosis (ADCP) effectively synergize antitumor therapy by phagocytes, even the contradictory relationship between antitumor effects and potential side effects can be balanced.^39^ Therefore, agents of CD47/SIRPα blockade for enhancement of phagocytosis function by blocking ‘don’t eat me’ signals combined with cancer-targeting therapeutic antibodies, FcR-mediated ADCC and ADCP, may be a more effective approach to potently eliminate tumors at the current stage.^12 34 40^

Previous studies have provided evidence that macrophages could effectively phagocytose tumor cells, and dendritic cells (DCs) could elicit increased activation of T cells in the tumor microenvironment after anti-CD47 therapy. Since DCs and macrophages are important components of innate immune system for phagocytosis and antigen presentation which can bridge innate and adaptive immunity, it is possible that they are synergistic to the removal of tumor cells, and to stimulate CD8^+^ T cells and initiate antitumor T-cell immune response.^41 42^ In the present study, the pep-20-mediated phagocytosis of tumors by macrophages initiated antitumor T-cell immune response with a marked increase of CD8^+^ T cells in tumor tissues, and the IFN-γ-secreting CD8^+^ T cells were significantly amplified.^43^ Macrophages engulfed OVA-expressing tumor cells and presented antigen, thus effectively activating OT-IT cell proliferation and secretion of IFN-γ cytokines in the presence of pep-20. In addition, removal of macrophages invalidated the therapeutic effects of pep-20 and impaired the activation of CD8^+^ T cells in tumor-bearing mice. Collectively, these results suggested that pep-20 depended on macrophages to exert antitumor effects and participated in motivating antitumor immune response.

A variety of therapeutic peptides and derivative drugs have been approved by the Food and Drug Administration for diverse disease diagnosis and treatments.^44^ Despite the limitations in wide applications of peptide drugs, such as low solubility, relatively short circulating T_1/2_, poor stability and limited oral bioavailability, many strategies including cyclization, PEGylation modifications, adjunction of D-amino acids and conjugation with serum albumin have been utilized to refine the peptide drugs.^45–47^Retro-inverso peptide ensures the stability of proteases which possess similar side-chain topologies and biological activities to interact with its receptor. However, pep-20 containing α-helices structure may not maintain the same biological activity as parent L-peptides to the retro-inverso isomerization due to the change at the secondary structure.^48^ D-amino acid substitutions at the N-terminal and C-terminal of the peptide which showed little effect on the α-helical structure were adopted to circumvent these problems.^49^ Pep-20-D12 containing D-amino acid substitutions at the N-terminal and C-terminal proved to be highly stable to enzymatic degradation and effectively maintain blocking activity.

RT results in inducing tumor cell death and triggers antitumor immune response, but in most cases, the response is insufficient to maintain, and relapses always occur.^28^ The combination of pep-20-D12 with IR treatment exerted more effectiveness in synergistically delaying tumor growth than IR alone, especially suppressing relapses. The effect of combining CD47 blockade with IR may be ascribed to multiple mechanisms. It is well accepted that IR can induce immunogenic cell death, resulting in pro-phagocytic signals exposure to the tumor cell membrane (calreticulin, phosphatidylserine and so on), which may be very conducive to enhancing macrophage-dependent phagocytosis of CD47 blockade. Moreover, our results showed that local IR leads to significant increase of intratumoral macrophages (CD11b^+^F480^+^) and monocytic MDSCs (CD11b^+^Ly6C^+^Ly6G^−^) which may differentiate into macrophages or directly participate in the phagocytosis of tumor cells.^50^

## Conclusion

In conclusion, a novel pep-20 peptide was identified by phage display bio-panning, which could markedly interfere the CD47/SIRPα interaction, enhance macrophage-mediated phagocytosis and elicit antitumor effects. The proteolysis-resistant analog pep-20-D12 could serve as a potential candidate for cancer immunotherapy by blocking CD47/SIRPα, especially in combination with RT to elicit synergistic effects.

10.1136/jitc-2020-000905.supp3 Supplementary data
