# Supplementary material for: CD47/SIRPα blocking peptide identification and synergistic effect with irradiation for cancer immunotherapy
Source: J Immunother Cancer. 2020 Oct 5;8(2):e000905. doi: 10.1136/jitc-2020-000905 (PMC7537338; doi:10.1136/jitc-2020-000905)
Supplement: Supplementary data [file jitc-2020-000905supp001.pdf]

## Supplementary Figures

### Figure S1

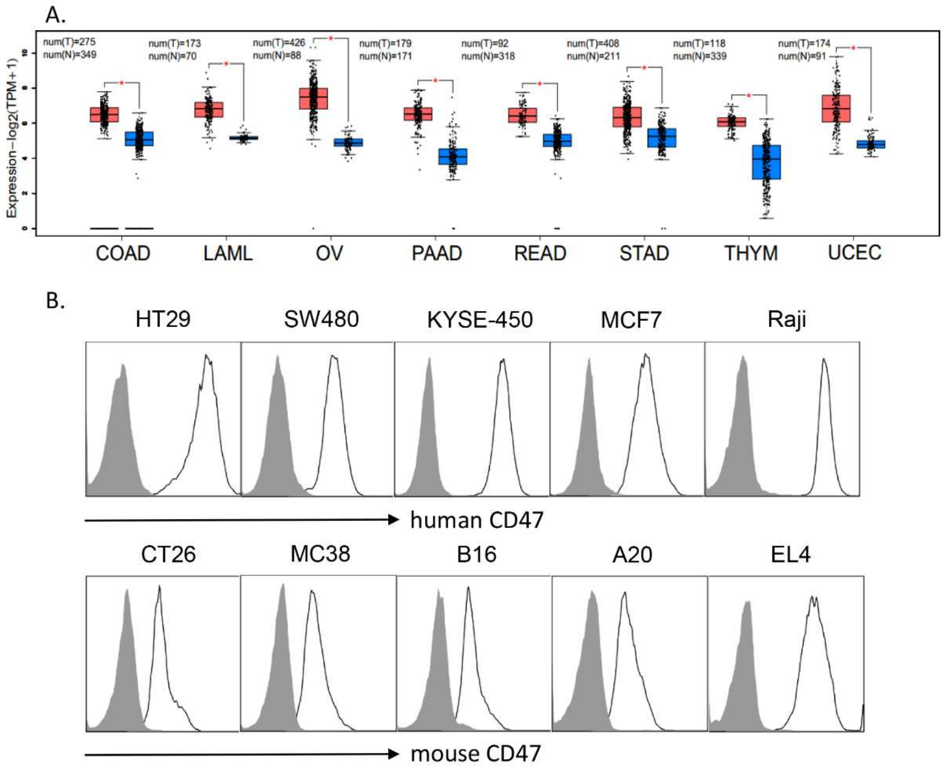


**Figure S1. CD47 is significantly overexpressed in tumors.** (A) Analysis of CD47 expression in human different types of tumor tissues and normal counterparts from TCGA and GTEx datasets were determined by GEPIA. **P* < 0.01. Colon Cancer (COAD); Acute Myeloid Leukemia (LAML); Ovarian Cancer (OV); Pancreatic Cancer (PAAD); Rectal Cancer (READ); Stomach Cancer (STAD); Thymoma (THYM); Endometrioid Cancer (UCEC); The Cancer Genome Atlas (TCGA); Genotype-Tissue Expression (GTEx). (B) Expression of CD47 on different tumor cell lines. CD47 expression on human or mouse tumor cell lines were detected by flow cytometry, the grey shades means matched isotype control.

**Figure S2**

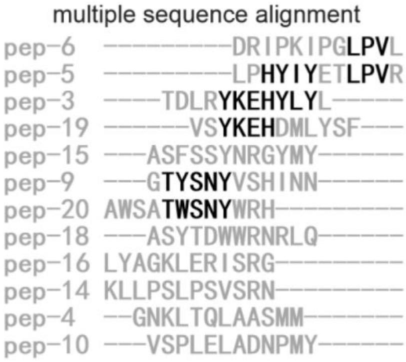


**Figure S2. Multiple sequence alignment of candidate peptides.** Peptide sequences were aligned using Clustal Omega after DNA sequencing, and the consensus sequence were labeled in bold.

**Figure S3**

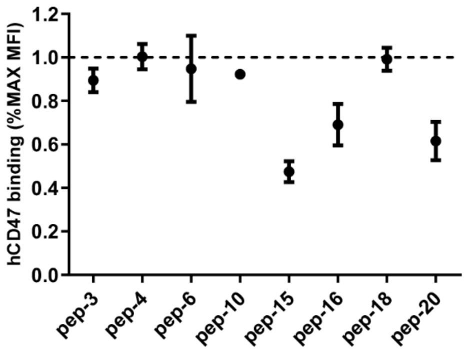


**Figure S3. Blocking activities of hCD47 binding peptides.** Flow cytometry analysis of hCD47 protein binding to CHO stably expressing human Sirpα cells in the presence of 200 μM candidate peptides. The data represented the mean fluorescence intensity normalized to the hCD47 protein binding without peptides. Error bars indicate standard deviation of triplicates. Data are represented as means ± SEM.

**Figure S4**

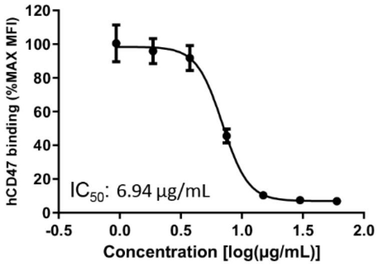


**Fig.S4. The blockade of anti-CD47 to CD47/Sirpα interaction.** Dose response curves of anti-human CD47 antibody (B6H12) interfering the human CD47/Sirpα interaction. Flow cytometry analysis of human CD47-IgV-Domain-hIg fusion protein binding to CHO stably expressing human Sirpα cells in the presence of B6H12 with varying gradient concentrations. The data represented as the mean fluorescence intensity normalized to the maximum binding. Data are represented as means ± SEM.

**Figure S5**

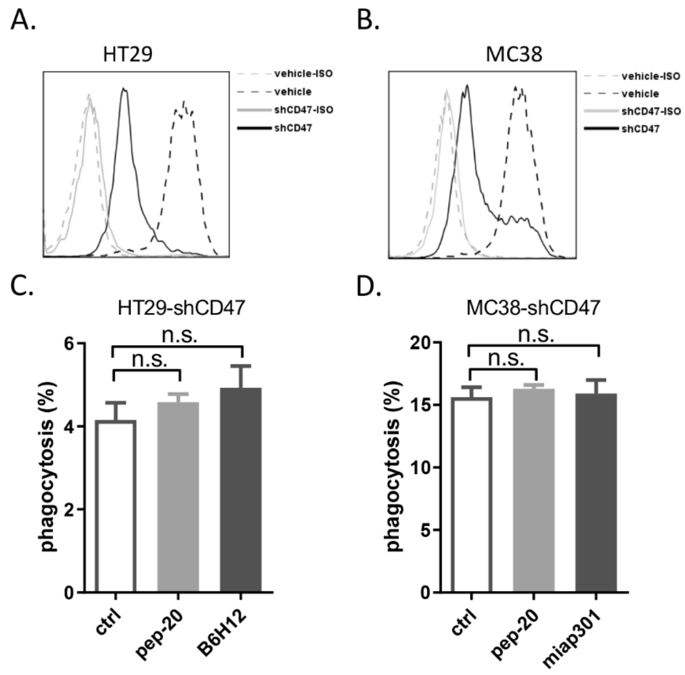


**Fig.S5. CD47 knockdown impairs the efficacy of pep-20 to induce macrophage-mediated phagocytosis of tumor cells.** (A, B) CD47 expression on HT29 (A) and MC38 (B) cells lines transfected shCD47 or vehicle was examined by flow cytometry. The gray histogram represents the matched isotype control. (C, D) Phagocytosis assays were performed by co-culture of CD47 knockdown $GFP^{+}$HT29 (C) and MC38 (D) cells with corresponding macrophages in the presence of PBS buffer, 100 μM pep-20, 20 μg/mL anti-human CD47 antibody (B6H12) or anti-mouse CD47 antibody (miap301) at a 1 ： 4 ratio in serum-free medium at 37℃ for 4 h. The percentage of $GFP^{+}$ macrophages in total macrophages were detected by flow cytometry. Data are represented as means ± SEM. Statistical significance was determined by unpaired Student's *t* test. **P* < 0.05; ***P* < 0.01; ****P* < 0.001.

**Figure S6**

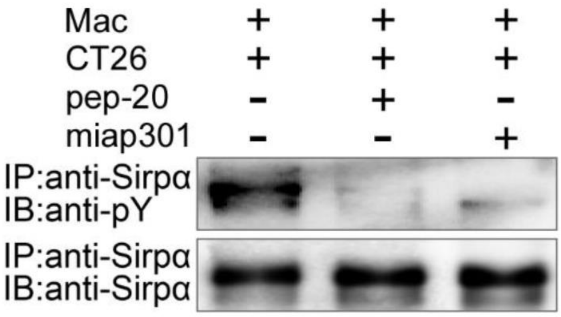


**Figure S6. Pep-20 reduces the tyrosine residues phosphorylation of Sirpα via CD47 engagement.** Mouse bone marrow-derived macrophages were incubated with CT26 cells at 37°C for 30 min in the presence of PBS (lane 1), 100 μM pep-20 (lane 2), 20 μg/mL of anti-mouse CD47 antibody (miap301, lane 3). The cell lysates were harvested for immunoprecipitation and immunoblotting. IP, immunoprecipitation; IB, immunoblotting; anti-pY, anti-posphotyrosine.

**Figure S7**

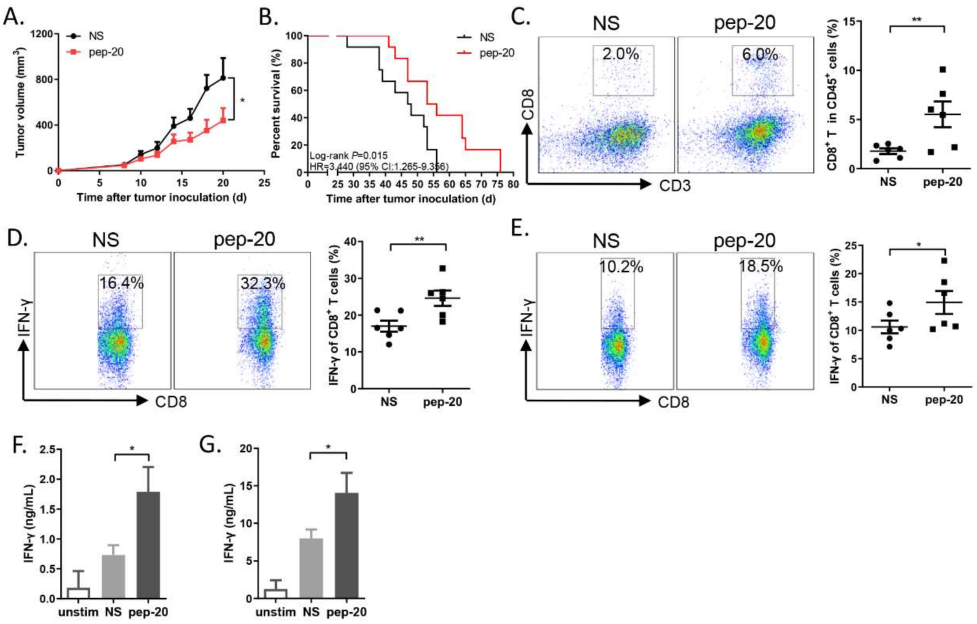

**Figure S7. Pep-20 inhibits the growth of tumors and activates antitumor T-cell immune response in CT26 tumor-bearing mice.** BALB/c mice were transplanted with 2×10$^5$ CT26 cells on the right flank, until the tumors volumes reached to around 50mm$^3$. (A) Mice were treated s.c. with 2 mg/kg of pep-20 or normal saline as the negative control at the peritumoral site every day for 2 weeks (*n* = 6). (B) Mice were treated with 2 mg/kg of pep-20 for 4 weeks and overall survival were monitored (*n* = 12). (C) Tumors were detected for the percentage of tumor-infiltrating CD8$^+$ T cells in total CD45$^+$ cells (*n* = 6). (D, E) Mice draining lymph nodes (D) and spleens (E) were obtained and stimulated with 20 ng/mL of PMA and 1 μM ionomycin containing protein transport inhibitor cocktail for 4 h. Frequencies of IFN-γ expressing CD8$^+$ T cells were detected by flow cytometry (*n* = 6). (F, G) Mice draining lymph nodes (F) and spleens (G) were obtained and stimulated with 0.5 μg/mL of anti-CD3 and 0.5 μg/mL of anti-CD28 antibodies for 3 days. Cellular supernatant from draining lymph nodes and spleens of IFN-γ secreting was measured with ELISA assay (*n* = 6). Data are represented as means ± SEM. Statistical significance was determined by unpaired Student's *t* test. *$P$ < 0.05; **$P$ < 0.01. Kaplan-Meier survival curves were evaluated by log-rank analysis.

**Figure S8**

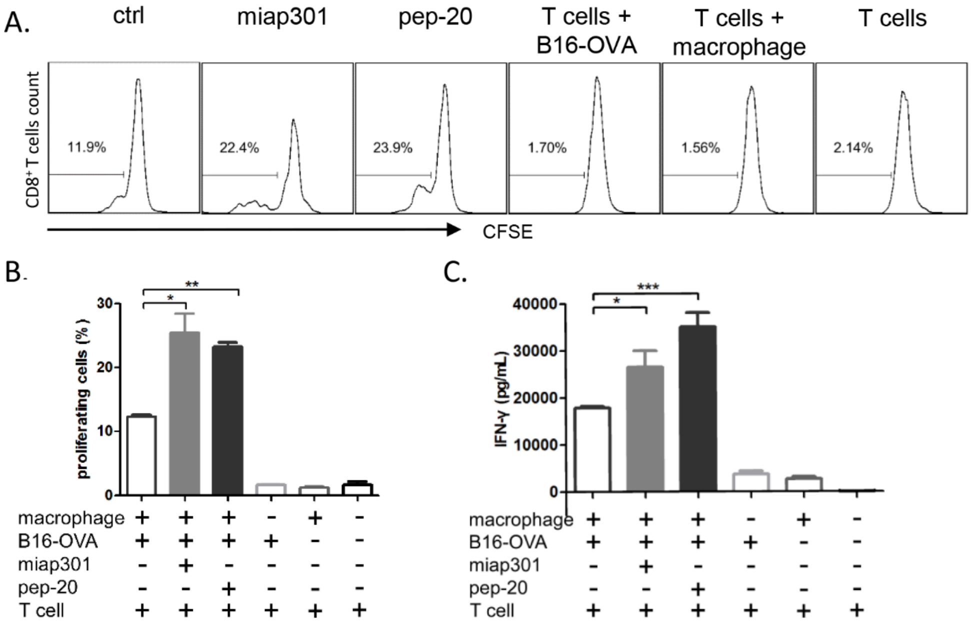


**Figure S8. Pep-20 enhances the activity of CD8⁺ T cells via macrophages.** (A, B) Bone marrow derived macrophages (BMDMs) were incubated with B16-OVA cells in the presence of PBS, 100 μM pep-20 or 20 μg/mL anti-mouse CD47 antibody (miap301) overnight. The peripheral lymph node cells, from OT-I TCR transgenic mice labeled with 0.5 μM CFSE, were co-cultured for 3 days. Cell proliferation were determined according to the percentage of $CFSE^+$ $CD8^+$ T cells by flow cytometry. (C) IFN-γ secreting of $CD8^+$ T cells were measured with ELISA assay. Data are represented as means ± SEM. Statistical significance was determined by unpaired Student's *t* test. *$P < 0.05$; **$P < 0.01$; ***$P < 0.001$.

**Figure S9**

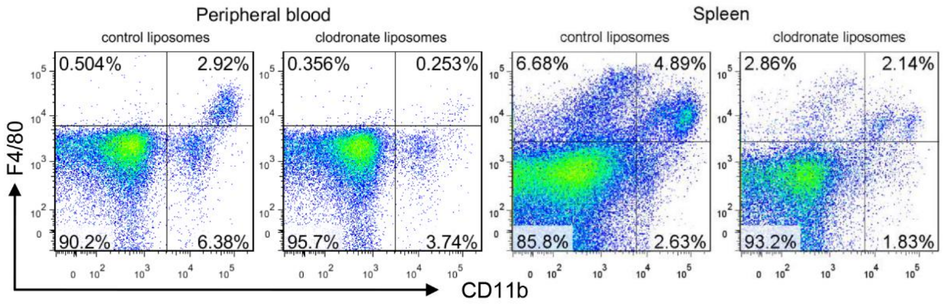


**Figure S9. Depletion of macrophages by clodronate liposome treatment *in vivo*.** For macrophage depletion, C57BL/6 mice were injected i.p. with 150 μL of clodronate liposomes or control liposomes, then peripheral blood and splenocytes were isolated from the mice 3 days later. The percentage of $CD45^+$ $CD11b^+$ $F4/80^+$ macrophages were evaluated by flow cytometry.

**Figure S10**

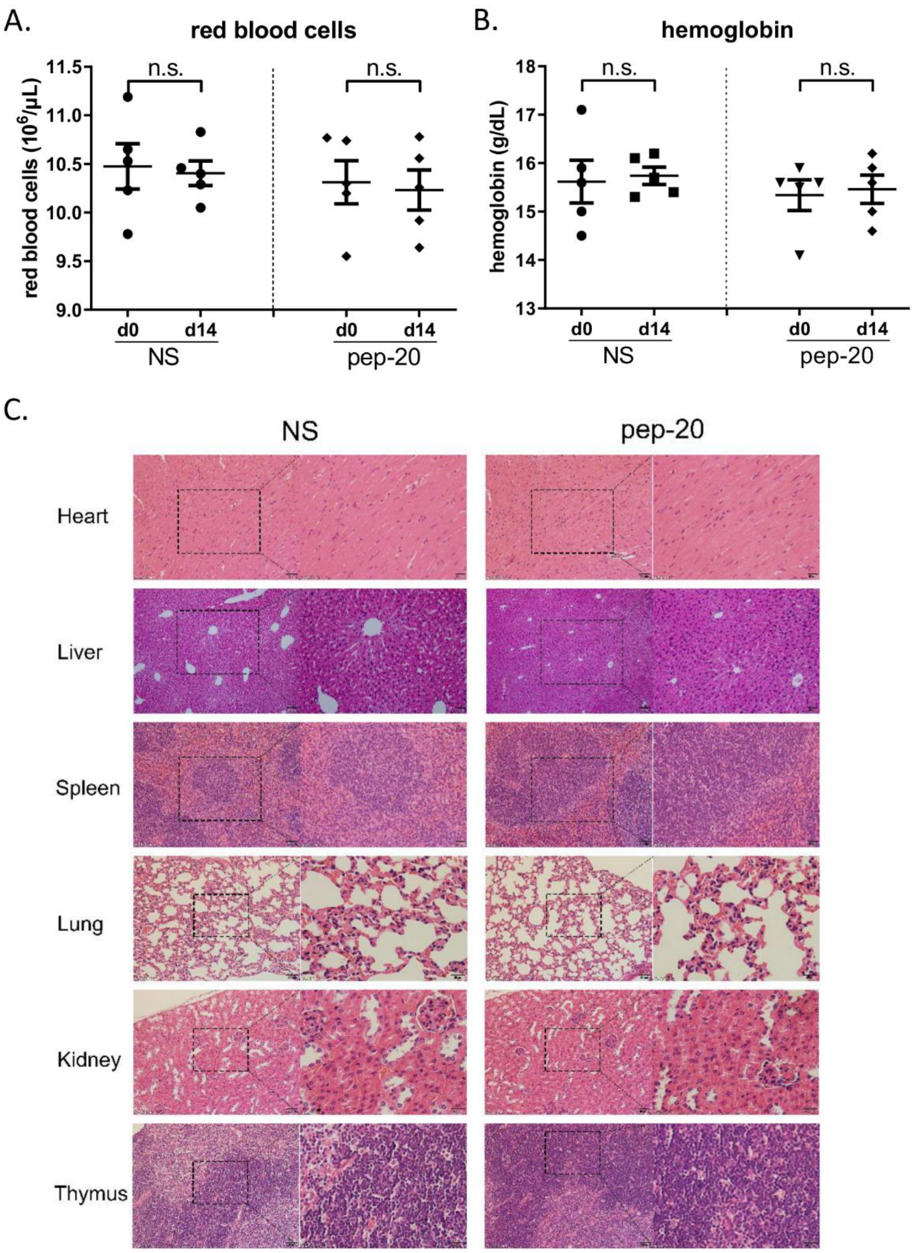


**Figure S10. Pep-20 displays no significant toxicity in mice.** (A, B) Hematological toxicity analysis. Normal C57BL/6 mice were injected s.c. with 2 mg/kg of pep-20 or normal saline as the negative control daily from day 1 to 14 (*n* = 5). On days 0 and 14, hematologic parameters were analyzed. Red blood cell count (A), hemoglobin level (B) have no significant difference between pep-20 group and control group. (C) Representative H&E staining images of pep-20 treated. Normal C57BL/6 mice were injected s.c. with 2 mg/kg of pep-20 or normal saline as the negative control daily for 14 days (*n* = 5). The mouse primary organs of heart, liver, spleen, lung, kidney and thymus

were stripped and tissue sections were processed for standard H&E staining. Representative images are shown. (Lift scale bar: 100 or 50 μm, Right scale bar: 20 μm). Data are represented as means ± SEM. Statistical significance was determined by unpaired Student's *t* test. n.s.: no significance.

**Figure S11**

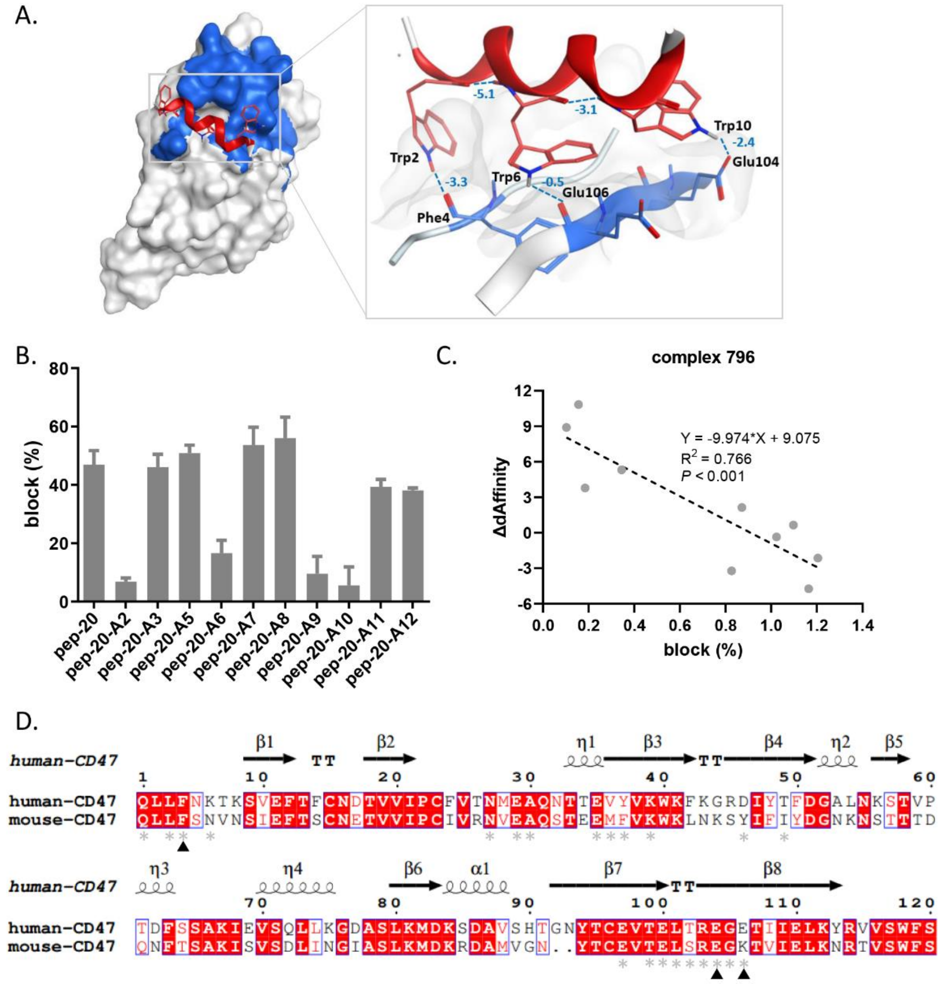


**Figure S11. Structure and docking model of pep-20 and CD47.** (A) Docking model of pep-20 and CD47. The interaction of pep-20 with human CD47 protein (PDB ID: 2JJS) was predicted by ZDOCK. Pep-20 shown in red ribbon, CD47 shown in gray surface, and the interaction domain of CD47-Sirpα shown in blue surface. (B) Blocking efficacy of pep-20 derived alanine mutation peptides. Flow cytometry analysis of hCD47 protein binding to CHO stably expressing human Sirpα cells in the presence of pep-20-based alanine mutation peptides. The data represented the mean fluorescence intensity normalized to the pep-20 blocking efficacy. Data are represented as means ± SEM. (C) Correlation analysis between the blocking rate and ΔdAffinity. Blocking rates were obtained from (B), and ΔdAffinity between pep-20 and alanine mutant peptides were calculated by the MOE software. (D) Alignment of human and mouse CD47 IgV domain sequences (UniProt accession no.Q61735 and no.Q08722). CD47 conserved residues were boxed and highlighted in red shaded. Residues of CD47 interacting with Sirpα were marked with asterisk and binding sites of pep-20 to hCD47 were indicated by bold triangular. Alignment was performed using the ESPript version 3.0 (http://espript.ibcp.fr/ESPript/cgi-bin/ESPript.cgi).

**Figure S12**

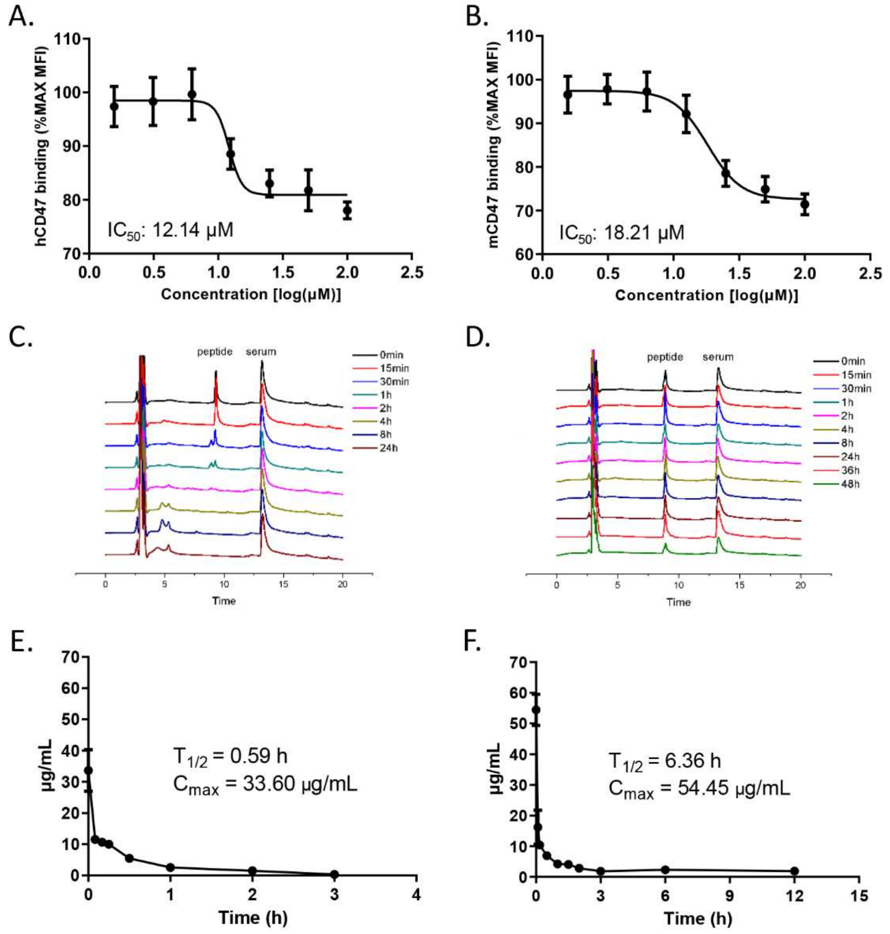


**Figure S12. Pep-20-D12 blocks the CD47-Sirpα interaction and resists to proteolysis compared with parent pep-20.** (A, B) Dose response curves of pep-20-D12 interfering CD47-Sirpα interaction. Human (A) or mouse (B) CD47-IgV-Domain-hIg fusion protein binding to CHO stably expressing human or mouse Sirpα cells in the presence of pep-20-D12 with varying gradient concentrations were analysis by flow cytometry. The data represented as the mean fluorescence intensity normalized to the maximum binding, and the sigmoidal dose-response curves were generated by GraphPad Prism. (C, D) Decomposition of pep-20 (C) and pep-20-D12 (D) in 10% human serum. The mixture of peptide and serum were analyzed by RP-HPLC. Error bars indicate standard deviation of triplicates. Data are represented as means ± SEM. (E, F) The pharmacokinetic analysis of pep-20 and pep-20-D12. The plasma concentration-time curve of pep-20 (E) and pep-20-D12 (F) after intravenous administration of 40 mg/kg in C57BL/6 mice (*n* = 4 - 8). Data are represented as means ± SEM.

**Figure S13**

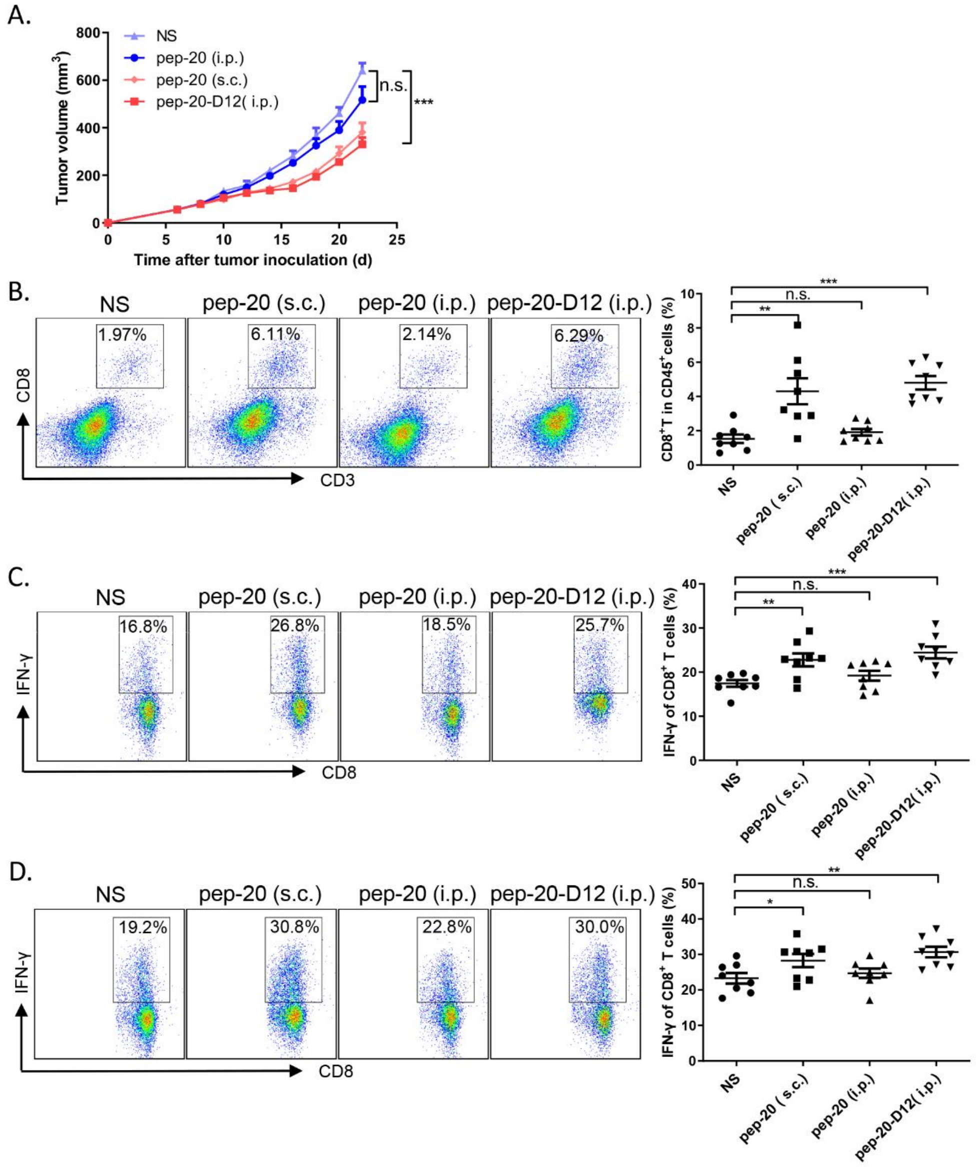


**Fig.S13. Pep-20-D12 has more effective antitumor efficacy compared with parent pep-20 via systemically treatment.** C57BL/6 mice were transplanted with $1\times10^6$ MC38 cells on the right flank, until the tumors volumes reached to around $50mm^3$. (A) Mice were treated with 2 mg/kg pep-20-D12 (i.p.), pep-20 (i.p. or s.c. of peritumoral ) for 2 weeks, normal saline as the negative control ($n = 8$). (B) Tumors were detected for the percentage of tumor-infiltrating $CD8^+$ T cells in total $CD45^+$ cells ($n = 8$). (C, D) Cells from mice draining lymph nodes (C) or spleens (D) were obtained and stimulated with 20 ng/mL of PMA and 1 µM ionomycin containing protein transport inhibitor cocktail for 4 h. Frequencies of IFN-γ expressing $CD8^+$ T cells were detected by flow cytometry ($n = 8$). Data are represented as means ± SEM. Statistical significance was determined by unpaired Student's *t* test. $^{**}P < 0.01$; $^{***}P < 0.001$. n.s.: no significance.

**Figure S14**

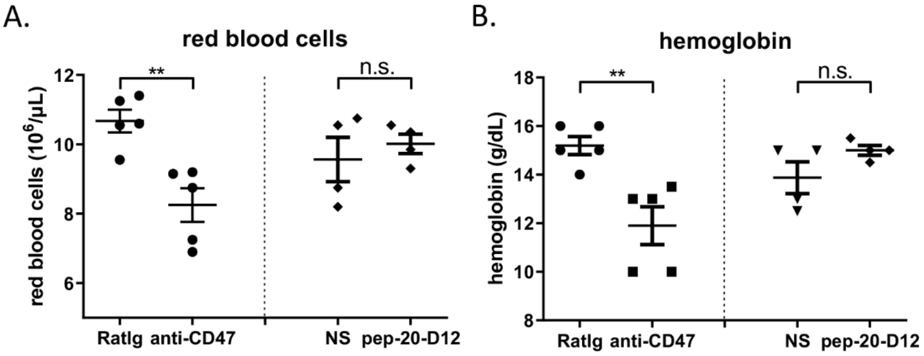


**Fig.S14. Pep-20-D12 displays no significant toxicity in mice compared with anti-CD47.** (A, B) MC38 tumor-bearing C57BL/6 mice were treated i.p. with 2 mg/kg pep-20-D12 every day for two weeks or 400μg anti-mouse CD47 antibody (miap301) every 3 days for a total of 5 times, and normal saline or ratIg as the negative controls, respectively (*n* = 4 - 5). On day 14, hematologic parameters of red blood cell (A), hemoglobin (B) level were analyzed. Data are represented as means ± SEM. Statistical significance was determined by unpaired Student's *t* test.

**Figure S15**

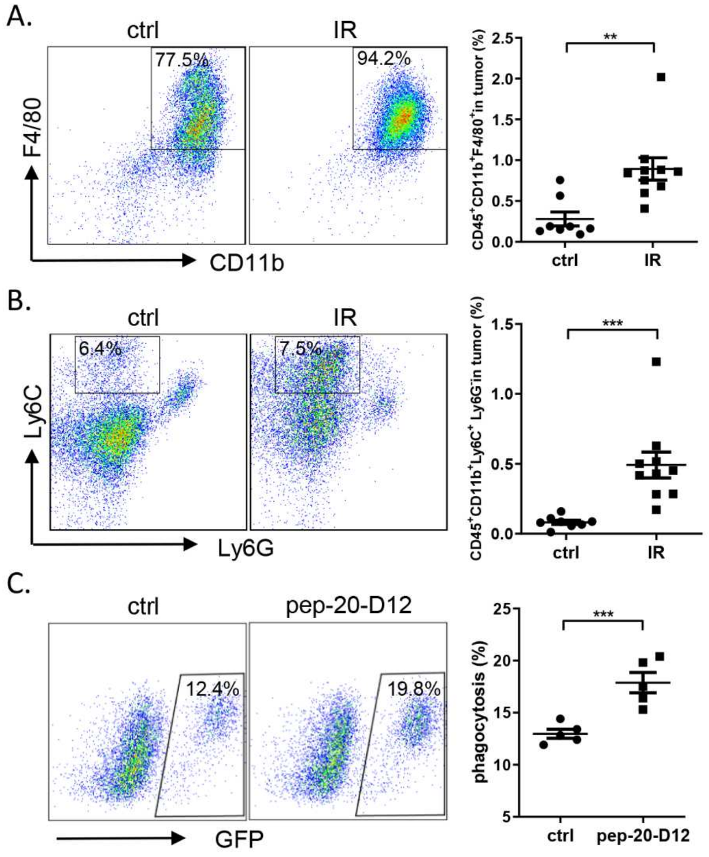


**Fig.S15. Irradiation-induced increases tumor-infiltrating macrophages and monocyte-derived MDSCs that can phagocytize tumor cells by pep-20-D12 treatment.** C57BL/6 mice were transplanted with $1\times10^6$ MC38 cells on the right flank, and tumors were locally received one 20 Gy dose IR after growed to reach ~100 $mm^3$. Tumor tissues were obtained 3 days later. (A, B) The percentages of tumor-infiltrating macrophages ($CD45^+CD11b^+F480^+$) (A) and monocyte-derived MDSCs ($CD45^+CD11b^+Ly6C^+Ly6G^-$) (B) in total tumor tissues were evaluated by flow cytometry ($n$ = 8 - 10). (C) Monocyte-derived MDSCs phagocytosis assays were performed by co-culture of tumor tissue cells with $GFP^+$ MC38 cells in the presence of 100 μM pep-20-D12 or PBS as negative control at 37℃ for 4 h. The percentage of $GFP^+$ monocyte-derived MDSCs in total M-MDSCs were detected by flow cytometry ($n$ = 5). Data are represented as means ± SEM. Statistical significance was determined by unpaired Student's $t$ test. $^*P < 0.05$; $^{**}P < 0.01$; $^{***}P < 0.001$.
