# Supplementary material for: CD47/SIRPα blocking peptide identification and synergistic effect with irradiation for cancer immunotherapy
Source: J Immunother Cancer. 2020 Oct 5;8(2):e000905. doi: 10.1136/jitc-2020-000905 (PMC7537338; doi:10.1136/jitc-2020-000905)
Supplement: Supplementary data [file jitc-2020-000905supp002.pdf]

Supplementary Tables

Table S1. Amino acid sequences and binding activity of selected peptides.

Name	Peptide sequence	Frequency (n/43)	K_D (μM)
3	TDLRYKEHYLYL	7	0.67±4.56
4	GNKLTQLAASMM	7	5.32±16.37
5	LPHYIYETLPVR	4	ND
6	DRIPKIPGLPVL	5	9.27±15.14
9	GTYSNYVSHINN	1	NA
10	VSPLELADNPMY	2	49.87±54.80
14	KLLPSLPSVSRN	2	>500
15	ASFSSYNRGYMY	2	14.73±20.04
16	LYAGKLERISRG	4	10.72±8.89
18	ASYTDWWRNRLQ	2	160.82±110.98
19	VSYKEHDMLYSF	1	NA
20	AWSATWSNYWRH	1	3.19±2.27

Peptides targeting hCD47 were obtained by bio-panning, the affinity of peptides to hCD47 was measured by the MST. NA means no available due to the poor solubility of peptides. ND means the K_D values failed to be detected despite the trend fluorescence changes.

Table S2. Organ coefficients analysis of pep-20-treated mice.

	Heart	Liver	Spleen	Lung	Kidney	thymus
NS	0.55±0.03	5.02±0.3	0.3±0.04	0.76±0.19	1.15±0.05	0.42±0.06
pep-20	0.58±0.05	5.01±0.16	0.31±0.3	0.77±0.18	1.14±0.06	0.36±0.07
P value	0.19	0.49	0.46	0.44	0.44	0.14

Normal C57BL/6 mice were injected s.c. with 2 mg/kg pep-20 or normal saline as the negative control daily for 14 days (n = 5). The mice organ tissues were stripped for organ coefficients analysis.

Table S3. Hepatic damage analysis of pep-20-treated mice.

	ALT	AST
NS	6.79±0.92	23.40±4.41
pep-20	7.03±0.86	28.41±5.08
P value	0.36	0.09

Normal C57BL/6 mice were injected s.c. with 2 mg/kg pep-20 or normal saline as the negative control daily for 14 days (*n* = 5). Mice serum samples were collected and hepatic damage analysis were performed for standard aspartate aminotransferase assay (AST) and alanine aminotransferase assay (ALT).

Table S4. Sequences of pep-20 modified with partial D-amino acid substitution both N- and C-terminal flanking regions.

Name	Peptide sequence	Blocking rate relative to pep-20 (%)
pep-20	AWSATWSNYWRH	100
pep-20-D2	a WSATWSNYWR h	83
pep-20-D3	a WSATWSNYW r h	75
pep-20-D4	a WSATWSNY w r h	96
pep-20-D6	a w SATWSNYWR h	NA
pep-20-D7	a w SATWSNYW r h	NA
pep-20-D8	a w SATWSNY w r h	NA
pep-20-D10	a w s ATWSNYWR h	NA
pep-20-D11	a w s ATWSNYW r h	NA
pep-20-D12	a w s ATWSNY w r h	105

Peptide sequences of lower-case characters represent D-amino acids. The blockade of peptides to hCD47 relative to parent pep-20 was measured. NA means no available due to the poor solubility or the blocking rate failed to be detected.
