# Supplementary material for: CD47/SIRPα blocking peptide identification and synergistic effect with irradiation for cancer immunotherapy
Source: J Immunother Cancer. 2020 Oct 5;8(2):e000905. doi: 10.1136/jitc-2020-000905 (PMC7537338; doi:10.1136/jitc-2020-000905)
Supplement: Supplementary data [file jitc-2020-000905supp003.pdf]

BMJ Publishing Group Limited (BMJ) disclaims all liability and responsibility arising from any reliance placed on this supplemental material which has been supplied by the author(s)

Supplementary Methods

Mice

C57BL/6 and BALB/c mice were purchased from the Beijing Vital River Laboratory, China. OT-I TCR transgenic mice were gifted from Prof. Xuanming Yang's lab (Shanghai Jiao Tong University, China). All the mice were housed in specific pathogen free conditions. All animal experiments were performed in accordance to the animal experimental guidelines and approved by the Ethics Committee of zhengzhou University.

Cell lines and cell culture

MC38 and MCF7 cells were cultured in DMEM medium (GIBCO, USA) with 10% fetal bovine serum, 100 U/mL penicillin, 100 μg/mL streptomycin. B16-OVA (B16 expressing cytoplasmic ovalbumin), Chinese hamster ovary (CHO) cells stably expressing human or mouse Sirpα, CT26, HT29, Jurkat and Raji cells were cultured in RPMI 1640 medium (GIBCO, USA) containing 10% fetal bovine serum, 100 U/mL penicillin and 100 μg/mL streptomycin. Stable recombinant tumor cell lines expressing green fluorescent protein (GFP) were generated by transduction using a pLVX puro HIV-based lentiviral vector engineered to stably express a GFP fusion protein. GFP^+ B16-OVA cell line was gifted from Prof. Xuanming Yang (Shanghai Jiao Tong University, China). B16-OVA was provided by Prof. Shengdian Wang's lab (Institute of Biophysics, Chinese Academy of Sciences, China). CD47 knockdown HT29 or MC38 cells

Wang H, *et al. J Immunother Cancer* 2020; 8:e000905. doi: 10.1136/jitc-2020-000905

were generated by transduction human or mouse shCD47 using a pSicoR-GFP HIV-based lentiviral vector. All cells were maintained in a humidified atmosphere at 37°C and 5% CO_2.

Primary antibodies

Anti-human CD14 (61D3), anti-mouse F4/80 (BM8), anti-mouse CD45 (30-F11), anti-mouse CD3 (17A2), anti-mouse CD8α (53-6.7), anti-mouse IFN-γ (XMG1.2), anti-human CD47 (B6H12) were purchased from eBioscience, USA. Anti-mouse CD47 (miap301) was a gift from Xiaojuan Liu's lab (Institute of Biophysics, Chinese Academy of Sciences, China) and then purchased from Bio-XCell, USA. Anti-mouse Sirpα (ab8120) was purchased from Abcam, UK.

Gene expression and survival analysis

The CD47 expression of tumorand normal tissues invarious types of tumors was analyzed by the online database Gene Expression Profiling InteractiveAnalysis (GEPIA) (http://gepia2.cancer-pku.cn) for significance discriminate analysis.

Phage libraries and bio-panning

Solution-phase panning with affinity bead capture was performed according to the manufacturer's protocol issued by the Ph.D.-12 Phage Display Peptide Library (New England BioLabs, USA). Solution panning improves accessibility

of the protein binding site for phage displayed peptides. Furthermore, the bio-panning began with the incubation of the phage library with both human CD47-IgV-Domain-hIg fusion proteins (ACRO Biosystems, USA) -protein A/G magnetic beads (Millipore, USA) complex or human Ig-protein A/G magnetic beads complex, peptides specific for human Ig and protein A/G magnetic beads are avoided by alternating rounds of panning. Bead-specific peptides can be avoided by alternating rounds between the matrix types. Briefly, primary phage (2×10^{11} pfu) were incubated with human Ig-protein A/G magnetic beads complex for 2 h on a rocker platform at 4°C, in which the phage is pre-incubated with the beads in the absence of target. The supernatant containing unbound phage was then collected and incubated with human CD47-IgV-Domain-hIg fusion proteins-protein A/G magnetic beads complex for another 2 h at 4°C, rotating. Subsequently, the beads were gently washed 5 - 10 times with Tris Buffered Saline with Tween (TBST), TBS containing 0.1%-0.5% of Tween 20. Then eluting bound phages from the protein surface by incubation with 1 mL elution buffer (0.2 M Glycine-HCl, pH 2.2) for 10 min at 4°C, the product was immediately neutralized with 150 μL Tris-HCl buffer (1 M, pH 9.0). The bound phages were amplified and tittered in Escherichia coli ER2738 culture at 37°C. The remaining phage was subjected to another 4 rounds of bio-panning. To obtain high binding phage, the stringency of screening was gradually improved during panning. The ratio of output and input phage numbers were calculated for each round and single

BMJ Publishing Group Limited (BMJ) disclaims all liability and responsibility arising from any reliance placed on this supplemental material which has been supplied by the author(s)

phage plaques were selected for DNA sequencing randomly.

DNA sequencing and peptide synthesis

After 5 rounds of bio-panning *in vitro*, 43 plaques were chosen randomly and amplified from the titration plates. DNA sequencing was determined by the Genewiz Biotechnology Co. Ltd. (Suzhou, China). The L-peptides were synthesized by Fmoc-based solid-phase peptide synthesis (SPPS) according to the standard protocol and purified by high performance liquid chromatography (HPLC) (Shimadzu, Japan). The L-peptides segments of candidates and alanine substituation mutants were synthesized by Fmoc-based solid-phase peptide synthesis (SPPS) according to the standard protocol, purified by RP-HPLC (Shimadzu, Japan) and analyzed by ESI-MS (Waters, USA). The L-peptides modified with partial D-amino acid substitution in the N- and C-terminal flanking regions were synthesized by Nanjing Yuan-peptide biotechnology Co. Ltd. (Nanjing, China).

Immunoprecipitation and immunoblot assays

Bone marrow-derived macrophages (BMDMs, 2×10^6) were starved overnight in serum-free medium, and incubated with CT26 cells (1×10^7) which were suspended in serum-free medium (containing 2 mM sodium pervanadate) at 37°C for 30 min. After rinsing with cold PBS, cells were lysed with ice-cold lysis buffer (50 mM TrisHCl (pH 7.5), 150 mM NaCl, 1% NP-40, 1 mM

phenylmethylsulfonyl fluoride (PMSF), 1% protease inhibitors and 2 mM sodium pervanadate). After centrifugation at 15,000 g for 10 min, supernates were mixed with mouse anti-human Sirpα antibodies and protein A/G magnetic beads at 4°C overnight. After rinsing twice with lysis buffer, the beads mixture was eluted by SDS loading buffer, followed by western blot according to a standard procedure. Rabbit anti-Sirpα (ab8120, Abcam, UK) and mouse anti-phosphotyrosine IgG (4G10, Millipore, USA) were used as primary antibodies. HRP-conjugated goat anti-rabbit IgG and HRP-conjugated goat anti-mouse IgG (Sangon Biotech, China) were used as secondary antibodies.

T-cell activation assays

Bone marrow-derived macrophages (BMDMs) were incubated with B16-OVA cells overnight in serum-free medium at 1：1 ratio in round-bottom 96-well plates. Peripheral lymph node cells from OT-I TCR transgenic mice were labeled with 1 μM CFSE (eBioscience, USA). The CFSE-labeled lymph nodes were then incubated with BMDMs and B16-OVA cells in RPMI 1640 medium supplemented with 10% FBS at 37°C, 5% CO_2 and humidified conditions for 3 days. Cells were stained with anti-mouse CD3 and anti-mouse CD8α (eBioscience, USA) for 30 min at 4°C. Cell proliferation of $CD8^+$ T cells were evaluated according to CFSE staining by flow cytometry and the IFN-γ secreting were quantified with ELISA assay.

BMJ Publishing Group Limited (BMJ) disclaims all liability and responsibility arising from any reliance placed on this supplemental material which has been supplied by the author(s)

Mouse toxicity assays

Normal C57BL/6 mice were injected s.c. with 2 mg/kg peptides or normal saline as the negative control daily from day 1 to 14 (*n* = 5). On days 0 and 14, mice blood samples were collected via retro-orbital bleed. For MC38 tumor-bearing mice, blood samples were collected after treatment (*n* = 4 - 5). Hematologic and blood biochemical parameters were analyzed by ADVIA 2120i Hematology Analyzer (Siemens, Germany). After injection of 14 days, organ coefficients analysis, hepatic damage analysis (aspartate aminotransferase (AST)/alanine aminotransferase (ALT)) were performed and mouse organs were stripped and tissue sections were processed for standard H&E staining.

Structure and docking model prediction

The 3D structure of pep-20 was predicted by pep-FOLD3. The complex of pep-20 and CD47 (PDB ID: 2JJS) was predicted by ZDOCK. The ΔdAffinity between pep-20 and pep-20-derived alanine substituation analogue peptides were calculated by Ala-scanning of molecular operating environment (MOE, Chemical Computing Group, CA), which was used to display the structure of pep-20 and docking model of pep-20 and CD47 including interaction of residues.

Peptide stability assays

BMJ Publishing Group Limited (BMJ) disclaims all liability and responsibility arising from any reliance placed on this supplemental material which has been supplied by the author(s)

The peptides of 200 μM were dissolved in normal saline then mixed with fresh human serum (10%, vol/vol). Peptides were incubated for 48 h at 37°C of metal bath and samples were obtained after 0min, 15min, 30min, 1h, 2h, 4h, 8h, 24h, 36h and 48h. The acetonitrile-glacial acetic acid mixture was utilized to terminate the enzymatic reaction. After centrifuged at 12000 rpm for 10 min, the supernatant was analyzed by RP-HPLC to quantify peptide hydrolysis.

Pharmacokinetic assays *in vivo*

C57BL/6 mice with weight of 18-20 g were administrated with a single intravenous dose of 40 mg/kg of peptides. Blood samples were collected and centrifuged at 8000 rpm for 10 min at the indicated intervals. Plasma was further mixed with 10% $HClO_4$ at a ratio of 2∶1 (v/v) and centrifuged at 16,000 rpm for 10 min. The supernatant was analyzed by RP-HPLC. The pharmacokinetic parameters of peptides were determined by noncompartmental analysis (NCA) using Phoenix WinNonlin software program 5.2.1 (Certara, Princeton, USA). The maximum plasma concentration C_{max} and elimination half-life ($T_{1/2}$) were calculated.

Wang H, *et al. J Immunother Cancer* 2020; 8:e000905. doi: 10.1136/jitc-2020-000905
